# Microscopical Justification of Solid-State Wetting and Dewetting

**DOI:** 10.1007/s00332-022-09783-z

**Published:** 2022-04-02

**Authors:** Paolo Piovano, Igor Velčić

**Affiliations:** 1 Politecnico di Milano, Dipartimento di Matematica, P.zza Leonardo da Vinci 32, 20133 Milan, Italy; 2 WPI c/o Research Platform MMM “Mathematics-Magnetism-Materials”, Fak. Mathematik, Univ. Wien, 1090 Vienna, Austria; 3 Faculty of Electrical Engineering and Computing, University of Zagreb, Unska 3, 10000 Zagreb, Croatia

**Keywords:** Island nucleation, Wetting, dewetting, Winterbottom shape, Discrete-to-continuum passage, $\Gamma$-Convergence, Atomistic models, Surface energy, Anisotropy, Adhesion, Capillarity problems, Crystallization, 49JXX, 82B24

## Abstract

The continuum model related to the *Winterbottom problem*, i.e., the problem of determining the equilibrium shape of crystalline drops resting on a substrate, is derived in dimension two by means of a rigorous discrete-to-continuum passage by $\Gamma$-convergence of atomistic models taking into consideration the atomic interactions of the drop particles both among themselves and with the fixed substrate atoms. As a byproduct of the analysis, effective expressions for the drop surface anisotropy and the drop/substrate adhesion parameter appearing in the continuum model are characterized in terms of the atomistic potentials, which are chosen of Heitmann–Radin sticky-disk type. Furthermore, a threshold condition only depending on such potentials is determined distinguishing the wetting regime, where discrete minimizers are explicitly characterized as configurations contained in an infinitesimally thick layer, i.e., the wetting layer, on the substrate, from the dewetting regime. In the latter regime, also in view of a proven conservation of mass in the limit as the number of atoms tends to infinity, proper scalings of the minimizers of the atomistic models converge (up to extracting a subsequence and performing translations on the substrate surface) to a bounded minimizer of the Winterbottom continuum model satisfying the volume constraint.

## Introduction

**Fig. 1:**
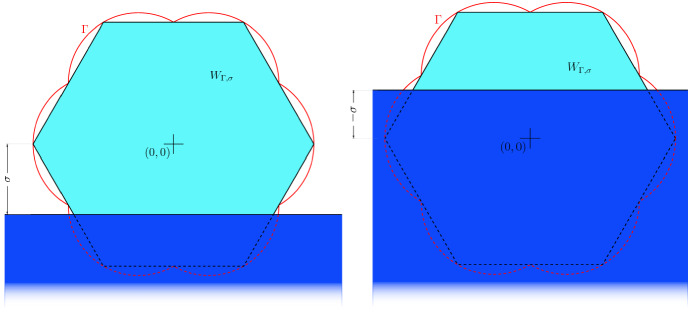
Winterbottom construction for the minimizer of ${\mathcal {E}}$, on the left for $\sigma >0$ and on the right for $\sigma <0$ (see Winterbottom 1967)

The problem of determining the equilibrium shape formed by crystalline drops resting upon a rigid substrate possibly of a different material is long standing in materials science and applied mathematics. The first phenomenological prediction of such shape for flat substrates is due to Winterbottom, who in (1967) designed what is now referred to as the *Winterbottom construction* (see Fig. 1) to minimize the drop surface energy in which both the *drop anisotropy* at the free surface and the *drop wettability* at the contact region with the substrate were taken into account (see (1)). The interplay between the drop material properties of anisotropy and wettability can induce different morphologies ranging from the spreading of the drops in a infinitesimally thick *wetting layer* covering the substrate, which is exploited, e.g., in the design of film coatings, to the nucleation of *dewetted islands*, that are solid-state clusters of atoms leaving the substrate exposed among them, which find other applications, such as for sensor devices and as catalysts for the growth of carbon and semiconductor nanowires (Jiang et al. 2016, 2017).

In this work, we introduce a discrete setting dependent on the atomistic interactions of drop particles both among themselves and with the substrate particles, and we characterize in terms of the parameters of the potentials governing such atomistic interactions the regime associated with the wetting layer, referred to in the following as the *wetting regime*. For the complementary parameter range, i.e., the *dewetting regime*, we microscopically justify the formation of solid-state dewetted islands by performing a rigorous discrete-to-continuum passage by means of showing the $\Gamma$-convergence of the atomistic energies to the energy considered in Jiang et al. (2016), Jiang et al. (2017) and by Winterbottom (1967).

In the continuum setting, the *Winterbottom problem* in Winterbottom (1967) essentially consists in an optimization problem based on an *a priori* knowledge of the surface anisotropy $\Gamma$ of the resting crystalline drop with the surrounding vapor, and of the *adhesivity* $\sigma$ related to the contact interface between the drop and the substrate. In the modern mathematical formulation in $\mathbb {R}^d$ for $d>1$, the energy associated with an admissible region $D\subset \mathbb {R}^d{\setminus }S$ occupied by the drop material, which is assumed to be a set of finite perimeter outside a fixed smooth substrate region $S\subset \mathbb {R}^d$, is given by

1 $$\begin{aligned} {\mathcal {E}}(D):=\int \limits _{\partial ^* D{\setminus }\partial S}\Gamma (\nu (\xi ))\, \mathrm {d}{\mathcal {H}}^{d-1}(\xi ) + \sigma {\mathcal {H}}^{1}(\partial ^* D\cap \partial S), \end{aligned}$$

where $\partial ^* D$ is the reduced boundary of *D*, $\nu$ is the exterior normal vector of *D*, and ${\mathcal {H}}^{d-1}$ the $(d-1)$-dimensional measure. The Winterbottom shape $W_{\Gamma ,\sigma }$ introduced in Winterbottom (1967) is defined as depicted in Fig. 1 by

$$\begin{aligned} W_{\Gamma ,\sigma }:=W_\Gamma \cap \{x\in \mathbb {R}^d\,:\, x_d\ge - \sigma \} \end{aligned}$$

where $W_\Gamma$ is the *Wulff shape*, i.e.,

$$\begin{aligned} W_\Gamma :=\{x\in \mathbb {R}^d\,:\, x\cdot \nu \le \Gamma (\nu ) \text { for every } \nu \in S^{d-1}\}. \end{aligned}$$

The Wulff shape $W_\Gamma$ is named after Wulff, who provided in (1901) its first phenomenological construction as the equilibrium shape for a free-standing crystal with anisotropy $\Gamma$ in the space (in the absence of a substrate or any other crystalline materials), and was afterward in Fonseca (1991) and Fonseca and Müller (1991) rigorously proved to be the unique minimum of (1) when $S=\emptyset$ in the presence of a volume constraint and after a proper scaling to adjust its volume (see also Taylor 1974, 1975).

The emergence of the Wulff and Winterbottom shapes has been already justified starting from discrete models in the context of statistical mechanics and the Ising model. We refer to the review (Dobrushin et al. 1992) (see also Ioffe and Schonmann 1998; Kotecký and Pfister 1994) for the 2-dimensional derivation of the Wulff shape in the scaling limit at low-temperature and to Bodineau et al. (2001), Pfister and Velenik (1996) and Pfister and Velenik (1997) for the setting related to the Winterbottom shape. More recently, the microscopical justification of the Wulff shape in the context of atomistic models depending on Heitmann–Radin sticky-disk type potentials (Heitmann and Radin 1980) has been addressed for $d=2$ and the triangular lattice in Au Yeung et al. (2012) by performing a rigorous discrete-to-continuum analysis by means of $\Gamma$-convergence. Subsequently, the deviation of discrete ground states in the triangular lattice from the asymptotic Wulff shape has been sharply quantified in Schmidt (2013) by introducing the $n^{3/4}$ law (see also Davoli et al. 2017), which has been then extended to the square lattice in Mainini et al. (2014a, 2014b), to the hexagonal lattice for graphene nanoflakes in Davoli et al. (2016), and to higher dimensions in Mainini et al. (2019) and Mainini and Schmidt (2020).

**Fig. 2:**
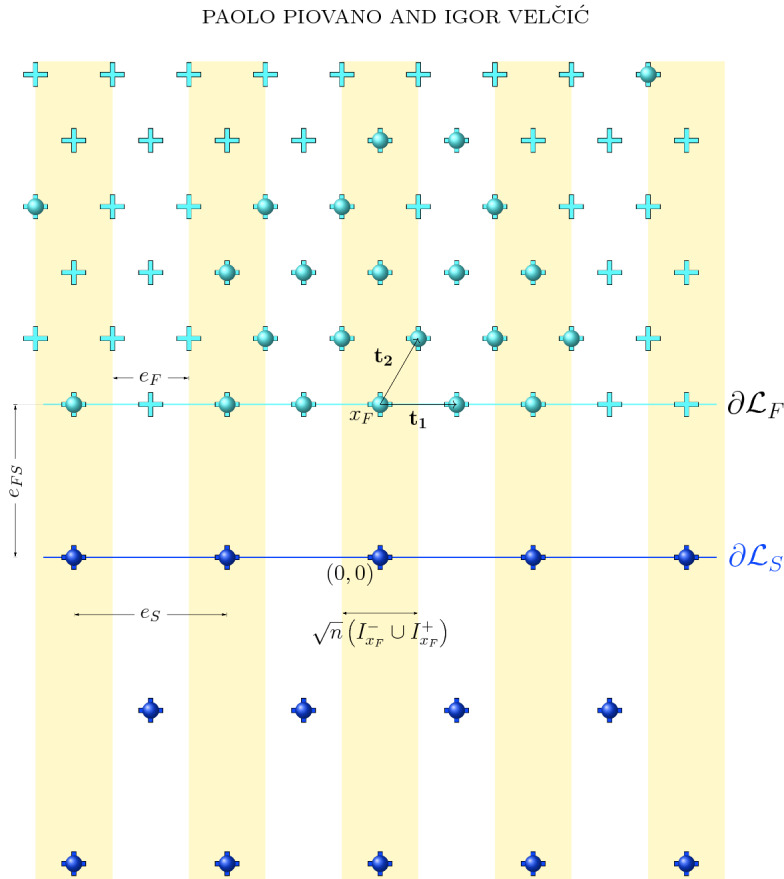
A portion of the lattices ${\mathcal {L}}_F$ and ${\mathcal {L}}_S$ is depicted with the respective lattice sites in light and dark blue crosses, respectively. The lattice ${\mathcal {L}}_S$ is fully occupied by substrate atoms represented by dark blue balls, while only some sites of ${\mathcal {L}}_F$ are occupied by film atoms represented by light blue balls. The “interface” $\partial {\mathcal {L}}_{F}$ consists of all the lattice sites on the light-blue line, while the “interface” $\partial {\mathcal {L}}_S$ consists of all the lattice sites on the dark-blue line. In yellow, we can see the *oscillatory set* related to the lattice sites in $\partial {\mathcal {L}}_{FS}$, which is introduced in Sect. 5

We intend here to generalize the analysis of Au Yeung et al. (2012) for $d=2$ to the situation of *S* being a half-plane by taking into account at the discrete level also the atomic interactions of the particles of the crystalline drops with the particles of the substrate, which we allow to possibly belong to a different species of particles, and we suppose occupying all sites of a fixed reference lattice ${\mathcal {L}}_S\subset S$. Film atoms are instead let free to move in a lattice ${\mathcal {L}}_F$ chosen to be triangular and contained in ${\mathbb {R}}^2{\setminus }{\overline{S}}$, so that admissible configurations of crystalline drops with $n\in \mathbb {N}$ film atoms are $D_n:=\{x_1,\ldots ,x_n\}\subset {\mathcal {L}}_F$ (see Fig. 2). By adding the contribution $E_S:({\mathbb {R}}^2{\setminus }{\overline{S}})^{n}\rightarrow \mathbb {R}\cup \{\infty \}$ to the energy of Au Yeung et al. (2012) to include atomic interactions of film atoms with substrate atoms, the overall energy $V_n$ of an admissible configuration $D_n:=\{x_1,\ldots ,x_n\}$ is given by

$$\begin{aligned} V_n(D_n)=V_n(x_1,\ldots ,x_n):=E_F(D_n)+E_S(D_n), \end{aligned}$$

where $E_F:({\mathbb {R}}^2{\setminus }{\overline{S}})^{n}\rightarrow \mathbb {R}\cup \{\infty \}$ represents the contribution of the atomic interactions among film atoms. More precisely, $E_F$ and $E_S$ are defined by

$$\begin{aligned} E_F(D_n)=E_F(x_1,\ldots ,x_n):=\sum _{i\ne j} v_{FF}(|x_i-x_j|) \end{aligned}$$

and

$$\begin{aligned} E_S(D_n)=E_S(x_1,\dots ,x_n):=\sum _{i=1}^n \sum _{s\in {\mathcal {L}}_S} v_\text {FS}(|x_i-s|), \end{aligned}$$

respectively, where $v_{F\alpha }$ for $\alpha =F,S$ are Heitmann–Radin sticky-disk two-body potentials attaining their minimum values $-c_\alpha$ at $e_{F\alpha }>0$, where $e_{FF}\equiv e_F$ is the distance between nearest neighbors in ${\mathcal {L}}_F$ and $e_{FS}$ is the distance between the lattices ${\mathcal {L}}_F$ and ${\mathcal {L}}_S$ (see Fig. 2 and Sect. 8 for a discussion on the positioning of the reference lattices). We recall that even with Heitmann–Radin potentials the crystallization of the minimizers of $V_n$ has been shown so far only in the case with $S=\emptyset$ in Heitmann and Radin (1980) by showing that the minimizers of $E_F$ are subset of a triangular lattice. The rigidity assumption of prescribing reference lattices ${\mathcal {L}}_F$ and ${\mathcal {L}}_S$, besides imposing the *non-interpenetration* for the film and substrate species of atoms, which remain separated by $\partial S$, also entails that the elastic energy associated with the mismatch between the optimal crystalline lattices of the two materials of the drop and the substrate at equilibrium is supposed to be all released by means of the *periodic dislocations* of the global reference lattice ${\mathcal {L}}:={\mathcal {L}}_F\cup {\mathcal {L}}_S$ prescribed at the film–substrate interface $\partial S$.

A study in which the complementary situation where elastic deformations of a homogeneous reference lattice ${\mathcal {L}}$ without dislocations between the film and the substrate are considered is available in Kreutz and Piovano (2019), where the linear-elastic models for epitaxially strained thin films introduced in Davoli and Piovano (2020), Davoli and Piovano (2019), Fonseca et al. (2007), Spencer (1999), and Spencer and Tersoff (1997) are derived from nonlinear elastic atomistic energies.

In our setting due to the periodic dislocations created at the interface between ${\mathcal {L}}_F$ and ${\mathcal {L}}_S$, the substrate interactions included in $E_S$ are in general non-constant (if not when $e_F$ is a multiple of $e_S$) and may result in *periodic oscillations* between null and negative contributions to the overall energy, referred to in the following as *periodic adhesion deficit*. The presence of such oscillations induces differences with the analysis carried out in Au Yeung et al. (2012) and could substantiate the employment of *homogenization techniques* for periodic structures (see Alberti and De Simone 2005 for the continuum setting). However, it then turns out that homogenization techniques are not needed as the “homogenized” limit actually coincides with the average in our setting (and in Caffarelli and Mellet 2007 for the continuum setting). Moreover, the periodic adhesion deficit at the drop/substrate region induces a *lack of compactness* for (the properly scaled) energy-equi-bounded sequences (even up to uniform translations), which is not treatable with only adopting local arguments at the substrate surface similar to the one employed in Au Yeung et al. (2012). In order to balance up the deficit, we subdivide drop configurations in *strips vertical to the substrate* so that enough boundary particles not adhering with the substrate (and so without deficit) are counted. Then, summing up all the strips allows to determine a *global lower bound* to the overall surface contribution and to recover compactness in a proper subclass of admissible configurations, i.e., *almost-connected configurations* (see Sect. 2.4), that are configurations which are unions of connected components positioned at “substrate bond” distance. Such limitation is then overcome by means of ensuring that mass does not escape on the infinite substrate surface.

Another reason for the lack of compactness with substrate interactions is the possibility for minimizing drop configurations to spread out on the infinite substrate surface forming an infinitesimal wetting layer, which for $E_S\not \equiv 0$ can be actually favored. Therefore, a peculiar aspect of our analysis resides in distinguishing such *wetting regime* from the *dewetting regime*. More precisely, we characterize a *dewetting threshold* in terms of the interatomic potentials $v_{FF}$ and $v_{FS}$, namely

2 $$\begin{aligned} {\left\{ \begin{array}{ll} c_{S}< 4c_{F} &{} \text {if }e_F\text { is a multiple of }e_{S},\\ c_{S}< 6c_{F} &{} \text {otherwise,} \end{array}\right. } \end{aligned}$$

under which the emergence of the minimizers of (1) with full $\mathbb {R}^2$-Lebesgue measure is shown.

The results of the paper are threefold (see Sect. 7): The first result, Theorem 2.2, is a crystallization result for wetting configurations achieved by induction arguments in which the dewetting threshold condition (2) is singled out by treating separately the situation of constant and non-constant substrate contributions. In this regard, notice that the characterization of the dewetting regime coming from continuum theories (see, e.g., Baer 2015) does not represent in general a good prediction for the discrete setting due to the deficit averaging effects taking place in the passage from discrete to continuum. More precisely, as described in Baer (2015) (with the extra presence of a gravity term perturbation of ${\mathcal {E}}$) the condition

3 $$\begin{aligned} \sigma >-\Gamma (\nu _S) \end{aligned}$$

is the natural requirement in the continuum “ensuring that it is not energetically preferred for minimizers to spread out into an infinitesimally thin sheet”. However, condition (3) coincides with the dewetting-threshold condition of the discrete setting only when $e_F$ is a multiple of $e_{F}$ (see 5 and 6), being otherwise the latter condition more restrictive.

The second result, Theorem 2.3, provides a *conservation of mass* for the solutions of the discrete minimum problems

4 $$\begin{aligned} \min _{D_n\subset {\mathcal {L}}_F}{V_n(D_n)}\ \end{aligned}$$

as the number *n* of atoms tends to infinity, which is crucial to overcome the lack of compactness outside the class of almost-connected sequences of energy-equibounded minimizers. In particular, it consists in proving that it is enough to select a connected component among those with largest cardinality for each solution of (4). This is achieved by proving compactness for almost-connected energy minimizers and then by defining a proper transformation ${\mathcal {T}}$ of configurations (based on iterated translations of connected components as detailed in Definition 2.1), which always allows to pass to an almost-connected sequence of minimizers.

The last result, Theorem 2.4, relates to the convergence of the minimizers of (4) as $n\rightarrow \infty$ to a minimizer of (1) in the family of crystalline drop regions

$$\begin{aligned} {\mathcal {D}}_\rho :=\{D\subset \mathbb {R}^2{\setminus }S\,:\, \text {set of finite perimeter, bounded and such that }|D|=1/\rho \}, \end{aligned}$$

whose existence follows also from the proof, where $\rho$ is the atom density in ${\mathcal {L}}_F$ per unit area.

Such convergence is obtained (up to extracting a subsequence and performing horizontal translations on the substrate *S*) as a direct consequence of the conservation of mass provided by Theorem 2.3 and of a $\Gamma$-convergence result for properly defined versions of $V_n$ and ${\mathcal {E}}$ in the space ${\mathcal {M}}(\mathbb {R}^2)$ of Radon measures on $\mathbb {R}^2$ with respect to the weak* convergence of measures as the number *n* of film atoms tends to infinity.

More precisely, we consider the one-to-one correspondence between drop configurations $D_n\subset {\mathcal {L}}_F$ and their associated empirical measures $\mu _{D_n}\in {\mathcal {M}}(\mathbb {R}^2)$ (see definition at (14)), introduce an energy $I_n$ defined on ${\mathcal {M}}(\mathbb {R}^2)$ such that

$$\begin{aligned} I_n(\mu _{D_n})=V_n(D_n), \end{aligned}$$

and prove the $\Gamma$-convergence of proper scalings $E_n$ of $I_n$, namely

$$\begin{aligned} E_n:=n^{-1/2}(I_n+6c_F n), \end{aligned}$$

with respect to the weak* convergence of measures, to a functional $I_\infty$ defined in such a way that

$$\begin{aligned} I_\infty \left( \rho \chi _D\right) ={\mathcal {E}}(D), \end{aligned}$$

for every set of finite perimeter $D\subset \mathbb {R}^2{\setminus }S$ with $|D|=1/\rho$ and for specific *effective expressions* of the surface tension $\Gamma$ and of the adhesivity $\sigma$ appearing in the definition (1) of ${\mathcal {E}}$ in terms of the interatomic potentials $v_{FF}$ and $v_{FS}$. In particular, we obtain that

5 $$\begin{aligned} \sigma :=2c_F-\frac{c_S}{q}, \end{aligned}$$

where *q* relates to the proportion between $e_F$ and $e_S$ (see 13), and $\Gamma (\nu (\cdot ))$ is found to be the $\pi /3$-periodic function such that

6 $$\begin{aligned} \Gamma (\nu (\varphi )):=2 c_F\left( \nu _2(\varphi )-\frac{\nu _1(\varphi )}{\sqrt{3}}\right) \end{aligned}$$

for every

$$\begin{aligned} \nu (\varphi )=\left( \begin{array}{c} -\sin \varphi \\ \cos \varphi \end{array} \right) \end{aligned}$$

with $$\varphi \in [0, \pi /3]$$.

A crucial difference with respect to Au Yeung et al. (2012) in the proof of the lower and upper bound of such $\Gamma$-convergence result is that the adhesion term in (1) can be negative and originates in view of the averaging of the periodic adhesion deficit related to the dislocations at the film–substrate interface. In particular, it is the limit of the adhesion portion of the boundary of auxiliary sets $H_n'$ associated with the configurations $D_n$ (see Definition 55 based on lattice *Voronoi cells*) in the *oscillatory sets* $O_n$ (see Fig. 2). We notice that for such averaging arguments extra care is needed, as the results available from the continuum theories cannot directly be applied to the auxiliary sets $H_n'$ when $e_F$ is not a multiple of $e_S$, e.g., with respect to Baer (2015) (see also Caffarelli and Mellet 2007) because of the non-constant deficit, and with respect to Alberti and De Simone (2005) when $4c_F\le c_S< 6c_F$ because of the discrepancy between the continuum and the discrete dewetting conditions.

All the results presented in the manuscript are obtained under the restrictive assumption of a fixed specific positioning of the film lattice ${\mathcal {L}}_F$ with respect to the substrate lattice ${\mathcal {L}}_S$, i.e., the closest atoms of ${\mathcal {L}}_F$ to ${\mathcal {L}}_S$ are positioned at a distance from at most one substrate atom given exactly by the constant at which the atomistic interaction potential between a film and a substrate atom attains its minimum, namely $e_{FS}$. This might be indeed not the optimal positioning in some situations, and in both (Piovano and Velčić 2021) and the forthcoming paper (Piovano and Velčić in preparation) we relax such assumption, both by showing how some other settings can be reduced to the model considered in this manuscript, and by introducing a few similar models (that will be shown to be treated with similar strategies to the ones presented in this manuscript) to which the missing situations can be reduced. We begin this analysis in the last section of the manuscript by listing some examples (see Examples 8.4, 8.5 and 8.6) of different relevant positioning of the reference lattices ${\mathcal {L}}_F$ and ${\mathcal {L}}_S$ that we show can be reduced to the model introduced in Sect. 2 thus, allowing us by Proposition 8.3 to recover all the main results of the manuscript, i.e., Theorems 2.2, 2.3, and 2.4 for such examples as well (and an example where we discuss the optimality of the positioning chosen in the model introduced in Sect. 2 for a particular choice of the lattice and atomistic parameters).

### Paper Organization

In Sect. 2, we introduce the mathematical setting with the discrete models (expressed both with respect to lattice configurations and to Radon measures) and the continuum model, and the three main theorems of the paper. In Sect. 3, we treat the wetting regime and prove Theorem 2.2. In Sect. 4, we establish the compactness result for energy-$E_n$-equibounded almost-connected sequences. In Sect. 5 we prove the lower bound of the $\Gamma$-convergence result. In Sect. 6, we prove the upper bound of the $\Gamma$-convergence result. In Sect. 7, we study the convergence of almost-connected transformations of minimizers and present the proofs of both Theorems 2.3 and 2.4. In Sect. 8, we present some other positioning of ${\mathcal {L}}_F$ and ${\mathcal {L}}_S$ that can be reduced to the setting introduced in Sect. 2.

## Mathematical Setting and Main Results

In this section, we rigorously introduce the discrete and continuous models, the notation, and definitions used throughout the paper, and the main results.

### Setting with Lattice Configurations

We begin by introducing a reference set ${\mathcal {L}}\subset \mathbb {R}^2$ for the atoms of the substrate and of the film, which we assume to remain separate. We define ${\mathcal {L}}:={\mathcal {L}}_S\cup {\mathcal {L}}_F$, where ${\mathcal {L}}_S\subset {\overline{S}}$ denotes the reference lattice for the substrate atoms, $S:=\mathbb {R}\times \{r\in \mathbb {R}\,:\, r<0\}$ is referred to as the *substrate region*, and ${\mathcal {L}}_F\subset \mathbb {R}^2{\setminus }{\overline{S}}$ is the reference lattice for the film atoms.

More precisely, we consider the substrate lattice as a fixed lattice, i.e., every lattice site in ${\mathcal {L}}_S$ is occupied by a substrate atom, such that

$$\begin{aligned} \partial {\mathcal {L}}_S:={\mathcal {L}}_S\cap \{(r,0):\,r\in \mathbb {R}\,\}=\{s_k:=(k e_S,0) \,:\,k\in \mathbb {Z}\} \end{aligned}$$

for a positive lattice constant $e_S$, and we refer to $\partial {\mathcal {L}}_S$ as to the *substrate surface* (or *wall*). For the film lattice ${\mathcal {L}}_F$, we choose a triangular lattice with parameter $e_F$ normalized to 1, namely

7 $$\begin{aligned} {\mathcal {L}}_F:=\{x_F+k_1 {\varvec{t}}_1+k_2 {\varvec{t}}_2\,:\, k_1\in \mathbb {Z}\text { and }k_2\in \mathbb {N}\cup \{0\} \} \end{aligned}$$

where

8 $$\begin{aligned} x_F:=(0,e_{FS})\end{aligned}$$

for a constant $$e_{FS}>0$$,

$$\begin{aligned} {\varvec{t}}_1:={1 \atopwithdelims ()0},\quad \text {and}\quad {\varvec{t}}_2:= \frac{1}{2}{1 \atopwithdelims ()\sqrt{3}}. \end{aligned}$$

We denote by $\partial {\mathcal {L}}_F$ the *lower boundary* of the film lattice, i.e.,

$$\begin{aligned} \partial {\mathcal {L}}_F:=\{x_F+k_1 {\varvec{t}}_1:\, k_1\in \mathbb {Z}\} \end{aligned}$$

and by $\partial {\mathcal {L}}_{FS}$ the collection of sites in the lower boundary of the film lattice at a distance of $e_{FS}$ from an atom in $\partial {\mathcal {L}}_S$, i.e.,

$$\begin{aligned} \partial {\mathcal {L}}_{FS}:= \partial {\mathcal {L}}_F \cap \left( \partial {\mathcal {L}}_S + e_{FS}{\varvec{t}}_3\right) \end{aligned}$$

where

$$\begin{aligned} {\varvec{t}}_3:={0 \atopwithdelims ()1} \end{aligned}$$

(see Fig. 2).

We refer to Sect. 8 for examples of other positioning of the reference lattices ${\mathcal {L}}_F$ and ${\mathcal {L}}_S$, which can be reduced to the one addressed in this mathematical setting. The sites of the film lattice are not assumed to be completely filled and we refer to a set of $n\in \mathbb {N}$ sites $x_1,\dots ,x_n\in {\mathcal {L}}_F$ occupied by film atoms as a *crystalline configuration* denoted by $D_n:=\{x_1,\dots ,x_n\}\subset {\mathcal {L}}_F$. Notice that the labels for the elements of a configuration $D_n$ are uniquely determined by increasingly assigning them with respect to a chosen fixed order on the lattice sites of ${\mathcal {L}}_F$. With a slight abuse of notation, we refer to $x\in D_n$ as an atom in $D_n$ (or in ${\mathcal {L}}_F$). We denote the family of crystalline configurations with *n* atoms by ${\mathcal {C}}_n$. Furthermore, given a set $A\subset {\mathbb {R}}^2$, its cardinality is indicated by $\#A$, so that

$$\begin{aligned} {\mathcal {C}}_n:=\{A\subset {\mathcal {L}}_F\,:\, \#A=n\}. \end{aligned}$$

For every atom $x\in {\mathcal {L}}_F$, we take into account both its atomistic interactions with other film atoms and with the substrate atoms, by considering the two-body atomistic potentials $v_{FF}$ and $v_{FS}$, respectively. We restrict to first-neighbor interactions and we define $v_{F\alpha }$ for $\alpha :=F,S$ as

9 $$\begin{aligned} v_{F\alpha }(r):={\left\{ \begin{array}{ll} +\infty &{}\text{ if } r<e_{F\alpha },\\ -c_{\alpha } &{}\text{ if } r=e_{F\alpha },\\ 0 &{} \text{ if } r>e_{F\alpha }, \end{array}\right. } \end{aligned}$$

with $$c_{\alpha }>0$$ and $$e_{FF}:=e_F=1$$.

In the following, we refer to *film* and *substrate neighbors* of an atom *x* in a configuration $D_n$ as to those atoms in $D_n$ at distance 1 from *x*, and to those atoms in ${\mathcal {L}}_S$ at distance $e_S$ from *x*, respectively. Analogously, we refer to *film* and *substrate bonds* of an atom *x* in a configuration $D_n$ as to those segments connecting *x* to its film and substrate neighbors, respectively. We also refer to the union of the closures of all film bonds of atoms in a configuration $D_n$ as the *bonding graph* of $D_n$, and we say that a crystalline configuration $D_n$ is *connected* if every *x* and *y* in $D_n$ are connected through a path in the bonding graph of $D_n$, i.e., there exist $\ell \le n$ and $x_k\in D_n$ for $k:=1,\dots ,\ell$ such that $|x_k-x_{k-1}|=1$, $x_1=x$, and $x_\ell =y$. Moreover, we define the *boundary of a configuration* $D_n\in {\mathcal {C}}_n$ as the set $\partial D_n$ of atoms of $D_n$ with less than 6 film neighbors. We notice here that with a slight abuse of notation, given a set $A\subset {\mathbb {R}}^2$ the notation $\partial A$ will also denote the topological boundary of a set $A\subset {\mathbb {R}}^2$ (which we intend to be always the way to interpret the notation when applied not to configurations in ${\mathcal {C}}_n$, or to lattices, such as for $\partial {\mathcal {L}}_{S}$, $\partial {\mathcal {L}}_{F}$, and $\partial {\mathcal {L}}_{FS}$).

The energy $V_n$ of a configuration $D_n:=\{x_1,\ldots ,x_n\}\subset {\mathcal {L}}_F$ of *n* particles is defined by

10 $$\begin{aligned} V_n(D_n)= V_n(x_1,\ldots ,x_n):=\sum _{i\ne j} v_{FF}(|x_i-x_j|)\,+\, E_S(x_1,\ldots ,x_n) \end{aligned}$$

where $E_S:(\mathbb {R}^2{\setminus }{\overline{S}})^{n}\rightarrow \mathbb {R}\cup \{\infty \}$ represents the overall contribution of the substrate interactions defined as

11 $$\begin{aligned} E_S(D_n)=E_S(x_1,\dots ,x_n):=\sum _{i=1}^n v^1(x_i), \end{aligned}$$

where the one-body potential $v^1$ is defined by

12 $$\begin{aligned} v^1(x):=\sum _{s\in {\mathcal {L}}_S} v_\text {FS}(|x-s|) \end{aligned}$$

for any $x\in \mathbb {R}\times \{r\in \mathbb {R}\,:\, r>0\}$. Notice that from the definition of $v_{FS}$ and $x_F$ for any $x\in {\mathcal {L}}_F$ the sum in (12) is finite and

$$\begin{aligned} v^1(x)\in \{0,-c_S\}. \end{aligned}$$

In the following, we will always focus on the case

13 $$\begin{aligned} e_S:=\frac{q}{p} \end{aligned}$$

for some $p,q\in \mathbb {N}$ without common factors, since the case of $e_S=re_F$ for some $r\in \mathbb {R}{\setminus }\mathbb {Q}$ is simpler, as the contribution of $E_S$ is negligible (namely, in this case $\# \partial {\mathcal {L}}_{FS}=1$). More precisely, for $e_S=re_F$ with $r\in \mathbb {R}{\setminus }\mathbb {Q}$ the same analysis (or the one in Au Yeung et al. (2012) applies, and, up to rigid transformations, minimizers converge to a Wulff shape in $\mathbb {R}^2{\setminus }S$ with the Wulff-shape boundary intersecting $\partial S$ at least in a point.

### Setting with Radon Measures

The $\Gamma$-convergence result is established for a version of the previously described discrete model expressed in terms of *empirical measures* since it is obtained with respect to the weak* topology of Radon measures (Ambrosio et al. 2000). We denote the space of Radon measures on $\mathbb {R}^2$ by ${\mathcal {M}}(\mathbb {R}^2)$, and we write $\mu _{n}{\mathop {\rightharpoonup }\limits ^{*}} \mu$ to denote the convergence of a sequence $\{\mu _n\}\subset {\mathcal {M}}(\mathbb {R}^2)$ to a measure $\mu \in {\mathcal {M}}(\mathbb {R}^2)$ with respect to the weak* convergence of measures. The empirical measure $\mu _{D_n}$ *associated with a configuration* $D_n:=\{x_1,\dots ,x_n\}\in {\mathcal {C}}_n$ is defined by

14 $$\begin{aligned} \mu _{D_n}:=\frac{1}{n} \sum _{i=1}^n \delta _{\frac{x_i}{\sqrt{n}}}, \end{aligned}$$

where $\delta _z$ represents the Dirac measure concentrated at a point $z\in \mathbb {R}^2$, and the family of empirical measures related to configurations in ${\mathcal {C}}_n$ is denoted by ${\mathcal {M}}_n$, i.e.,

15 $$\begin{aligned} {\mathcal {M}}_n:=\{\mu \in {\mathcal {M}}(\mathbb {R}^2)\ :\ \text {there exists } D_n\in {\mathcal {C}}_n\text { such that }\mu =\mu _{D_n}\}. \end{aligned}$$

The functional $I_n$ associated with the configurational energy $V_n$ and expressed in terms of Radon measures is given by

16 $$\begin{aligned} I_n(\mu ):= \left\{ \begin{array}{ll} \int _{({\mathbf {R}}^2{\setminus }{\overline{S}})^2 \backslash \text {diag}} n^2 v_{FF}(n^{1/2}|x-y|) d \mu (x) \otimes d \mu (y) &{} \quad \text {if }\mu \in {\mathcal {M}}_n, \\ \qquad \qquad \qquad + \int _{{\mathbf {R}}^2{\setminus }{\overline{S}}} n v^1 (n^{1/2}x) d \mu (x) &{}\quad \\ +\infty &{}\quad \text {otherwise,} \end{array}\right. \end{aligned}$$

where

$$\begin{aligned} \text {diag}:=\{(y_1,y_2) \in \mathbb {R}^2: y_1=y_2\}. \end{aligned}$$

We notice that the two versions of the discrete model are equivalent, since

17 $$\begin{aligned} V_n(D_n)=I_n(\mu _{D_n}) \end{aligned}$$

for every configuration $D_n\in {\mathcal {C}}_n$, where $\mu _{D_n}\in {\mathcal {M}}_n$ is defined by (14), and that $D_n$ minimizes $V_n$ among crystalline configurations in ${\mathcal {C}}_n$ if and only if $\mu _{D_n}$ minimizes $I_n$ among Radon measures of ${\mathcal {M}}(\mathbb {R}^2)$.

### Local and Strip Energies

We define a *local energy* $E_{\mathrm{loc}}$ per site $x\in {\mathcal {L}}_F$ with respect to a configuration $D_n$, by

18 $$\begin{aligned} E_{\mathrm{loc}}(x):={\left\{ \begin{array}{ll} \sum _{y\in D_n{\setminus }\{x\}} v_{FF}(|x-y|) \,+\,6c_F &{}\text {if }x\in D_n,\\ 0 &{}\text {if }x\notin D_n, \end{array}\right. } \end{aligned}$$

which corresponds in the case of an atom $x\in D_n$ to the number of missing film bonds of *x*. We also refer to deficiency $E_\mathrm{def}(x)$ of a site $x\in {\mathcal {L}}_F$ with respect to a configuration $D_n$ as to the quantity

19 $$\begin{aligned} E_{\mathrm{def}}(x):= {\left\{ \begin{array}{ll} E_{\mathrm{loc}}(x)\,+\,v^1(x) &{}\text {if }x\in D_n,\\ 0 &{}\text {if }x \notin D_n. \end{array}\right. } \end{aligned}$$

Furthermore, we define the *strip* ${\mathcal {S}}(x)$ associated to any lattice site $x:=(x^1,e_{FS} )\in D_n\cap \partial {\mathcal {L}}_{FS}$ with $x_1\in \mathbb {R}$ as the collection of atoms

20 $$\begin{aligned} {\mathcal {S}}(x)={\mathcal {S}}_{D_n}(x):=\{x,x_{\pm },{\tilde{x}},{\tilde{x}}_{\pm }\}\cap D_n \end{aligned}$$

where $x_{\pm }$, ${\tilde{x}}$, and ${\tilde{x}}_{\pm }$ are defined by

$$\begin{aligned}&x_{\pm }:=x\pm {\varvec{t}}_1,\\&{\tilde{x}}:=(x^1,y_M)\quad \text {where}\quad y_M:=\max \{y\ge 0\,:\, (x^1,y)\in D_n\},\\&{\tilde{x}}_{+}:={\tilde{x}}+{\varvec{t}}_2,\\&{\tilde{x}}_{-}:={\tilde{x}}+{\varvec{t}}_2-{\varvec{t}}_1\end{aligned}$$

(see Fig. 3).

**Fig. 3:**
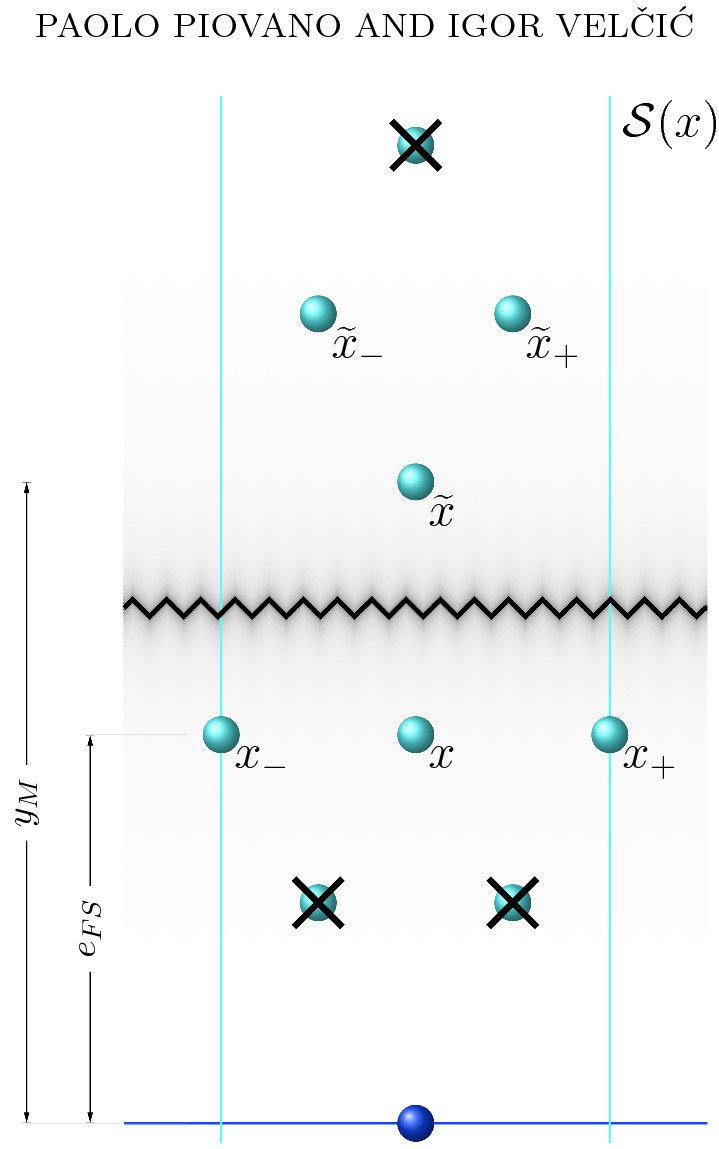
Strip ${\mathcal {S}}(x)$ centered at an atom $x\in \partial {\mathcal {L}}_{FS}$ of a crystalline configuration $D_n$ is depicted as an example of a strip containing all the elements $x,x_{\pm },{\tilde{x}},{\tilde{x}}_{\pm }$ with the possibility of the *strip center* *x* and the *strip top* ${\tilde{x}}$ to coincide if $y_M=e_{FS}$. The sites indicated by crossed atoms are sites of the planar lattice $\{x_F+k_1 {\varvec{t}}_1+k_2 {\varvec{t}}_2\,:\, k_1, k_2\in \mathbb {Z}\}$ that surely are not in $D_n$ by definition of ${\mathcal {L}}_F$ and ${\mathcal {S}}(x)$

In the following, we refer to *x* as the *strip center* of $${\mathcal {S}}(x)$$, to $$x_{\pm }$$ as the *strip lower (right and left) sides*, to $${\tilde{x}}$$ as the *strip top*, and to $${\tilde{x}}_{\pm }$$ as the *strip above (right and left) sides*. Note that *x* and $${\tilde{x}}$$ coincide if $$y_M=e_{FS} $$.

We define the *strip energy* associated with a strip ${\mathcal {S}}(x)$ by

21 $$\begin{aligned} E_{\mathrm{strip}}(x):= E_{\mathrm{strip, below}}(x)\,+\,E_{\mathrm{strip, above}}({x}), \end{aligned}$$

where

22 $$\begin{aligned} E_{\mathrm{strip, below}}(x):=&E_{\mathrm{loc}}(x)\,+\, \frac{1}{2} E_\mathrm{loc}(x_{+})\,+\, \frac{1}{2} E_{\mathrm{loc}}(x_{-})-c_S \end{aligned}$$

in the case $q\ne 1$, while

23 $$\begin{aligned} E_{\mathrm{strip, below}}(x):=&\frac{1}{2} E_{\mathrm{loc}}(x)\,+\, \frac{1}{4} E_\mathrm{loc}(x_{+})\,+\, \frac{1}{4} E_{\mathrm{loc}}(x_{-})-c_S \end{aligned}$$

in the case $$q= 1 $$, and

24 $$\begin{aligned} E_{\mathrm{strip, above}}(x):={\left\{ \begin{array}{ll} E_{\mathrm{loc}}({\tilde{x}})\,+\, w_+({\tilde{x}}) E_\mathrm{loc}({\tilde{x}}_{+})\,+\, w_-({\tilde{x}}) E_{\mathrm{loc}}({\tilde{x}}_{-}) &{}\text {if }{\tilde{x}}\ne x,\\ w_+({\tilde{x}}) E_{\mathrm{loc}}({\tilde{x}}_{+})\,+\, w_-({\tilde{x}}) E_\mathrm{loc}({\tilde{x}}_{-}) &{}\text {if }{\tilde{x}}=x \end{array}\right. } \end{aligned}$$

with weights $w_{\pm }({\tilde{x}})\in \{1/2,1\}$ given by25

### Almost-Connected Configurations

We recall from Sect. 2.1 that a configuration $D_n$ is said to be connected if every *x* and *y* in $D_n$ are connected through a path in the bonding graph of $D_n$, i.e., there exist $\ell \le n$ and $x_k\in D_n$ for $k:=1,\dots ,\ell$ such that $|x_k-x_{k-1}|=1$, $x_1=x$, and $x_\ell =y$, and we refer to maximal bonding subgraphs of $D_n$ connected through a path as *connected components* of $D_n$.

In order to treat the situation when $q\ne 1$, we need to introduce also a weaker notion of connectedness of configurations, which depends on $e_S$: We say that a configuration $D_n$ is *almost connected* if it is connected when $q=1$, and, if there exists an enumeration of its $k:=k_{D_n}$ connected components, say $D_n^i$, $i=1,\dots ,k$, such that each $D_n^{i}$ is separated by at most *q* from $\cup _{l=1}^{i-1}D_n^l$ for every $i=2,\dots ,n$, when $q\ne 1$.

We say that a family of connected components of $D_n$ form an *almost-connected component* of $D_n$ if their union is almost connected and, if $q\ne 1$, it is distant from all other components of $D_n$ by more than *q*.

#### Definition 2.1

Given a configuration $D_n\in {\mathcal {C}}_n$, we define the *transformed configuration* ${\mathcal {T}}(D_n)\in {\mathcal {C}}_n$ of $D_n$ as

$$\begin{aligned} {\mathcal {T}}(D_n):={\mathcal {T}}_2({\mathcal {T}}_1(D_n)), \end{aligned}$$

where ${\mathcal {T}}_1(D_n)$ is the configuration resulting by iterating the following procedure, starting from $D_n$:

- If there are connected components without any activated bond with an atom of $\partial {\mathcal {L}}_S$, then select one of those components with lowest distance from $\partial {\mathcal {L}}_S$;
- Translate the component selected at the previous step of a vector in direction $-{\varvec{t}}_2$ till either a bond with another connected component or with the substrate is activated.

(notice that the procedure ends when all connected components of ${\mathcal {T}}_1(D_n)$ have at least a bond with $\partial {\mathcal {L}}_S$), and ${\mathcal {T}}_2({\mathcal {T}}_1(D_n))$ is the configuration resulting by iterating the following procedure, starting from ${\mathcal {T}}_1(D_n)$:

- If there are more than one almost-connected component, then select the almost-connected component whose leftmost bond with $\partial {\mathcal {L}}_S$ is the second (when compared with the other almost-connected components) starting from the left;
- Translate the almost-connected component selected at the previous step of a vector $-kq{\varvec{t}}_1$ for some $k \in {\mathbf {N}}$ till, if $q=1$, a bond with another connected component is activated, or, if $q\ne 1$, the distance with another almost-connected component is less or equal to *q*;

(notice that the procedure ends when ${\mathcal {T}}_2(D_n)$ is almost connected).

We notice that the transformed configuration ${\mathcal {T}}(D_n)$ of a configuration $D_n\in {\mathcal {C}}_n$ satisfies the following properties:

(i) ${\mathcal {T}}(D_n)$ is almost connected;
(ii) Each connected component of ${\mathcal {T}}(D_n)$ includes at least an atom bonded to $\partial {\mathcal {L}}_S$;
(iii) $V_n({\mathcal {T}}(D_n))\le V_n(D_n)$ (as no active bond of $D_n$ is deactivated by performing the transformations ${\mathcal {T}}_1$ and ${\mathcal {T}}_2$);

and, if $D_n$ is a minimizer of $V_n$ in ${\mathcal {C}}_n$, then

(iv) $${\mathcal {T}}_1(D_n)= D_n$$;
(v) ${\mathcal {T}}$ consists of translations of the almost-connected components of $D_n$ with respect to a vector (depending on the component) in the direction $-{\varvec{t}}_1$ with norm in $\mathbb {N}\cup \{0\}$.

Finally, we also observe that the definitions of ${\mathcal {T}}_1$, ${\mathcal {T}}_2$, and ${\mathcal {T}}$ are independent from *n*.

### Continuum Setting

For every set of finite perimeter $D\subset \mathbb {R}^2{\setminus }S$, we define its anisotropic surface energy ${\mathcal {E}}$ by

26 $$\begin{aligned} {\mathcal {E}} (D):= \int _{\partial ^* D \backslash \partial S } \Gamma (\nu _D) d {\mathcal {H}}^1+\left( 2c_F-\frac{c_S}{q}\right) {\mathcal {H}}^1(\partial ^*D \cap \partial S) \end{aligned}$$

where $\partial ^* D$ denotes the reduced boundary of *D* and the *anisotropic surface tension* $\Gamma :{\mathbb {S}}^{1} \rightarrow {\mathbb {R}}$ is the function such that it holds

27 $$\begin{aligned} \Gamma (\nu (\varphi ))=2 c_F\left( \nu _2(\varphi )-\frac{\nu _1(\varphi )}{\sqrt{3}}\right) \end{aligned}$$

for every

$$\begin{aligned} \nu (\varphi )=\left( \begin{array}{c} -\sin \varphi \\ \cos \varphi \end{array} \right) \in {\mathbb {S}}^{1}\quad \text {with}\quad \varphi \in \left[ 0, \frac{\pi }{3}\right] , \end{aligned}$$

and $\Gamma \circ \nu$ is extended periodically on $\mathbb {R}$ as a $\pi /3$-periodic function. Notice that $\Gamma (\pm {\varvec{t}}_3)=2c_F$. By extending $\Gamma$ by homogeneity we obtain a convex function, and in particular a Finsler norm on $\mathbb {R}^2$.

We also use the following *auxiliary surface energy* depending on *n* in the proofs

28 $$\begin{aligned} {\mathcal {E}}_n (D):= \int _{\partial ^* D \cap (\mathbb {R}^2{\setminus }\overline{S_n})} \Gamma (\nu _D) d {\mathcal {H}}^1+\left( 2c_F-\frac{c_S}{q}\right) {\mathcal {H}}^1(\partial ^*D \cap \partial S_n) \end{aligned}$$

where

29 $$\begin{aligned} S_n:=S+\frac{e_{FS} }{\sqrt{n}}{\varvec{t}}_3. \end{aligned}$$

### Main Results

In this section, the rigorous statements of the main theorems of the paper are presented. We begin with the following result that characterizes the wetting regime in terms of a condition only depending on $v_{FF}$ and $v_{FS}$, and the minimizers in such regime.

#### Theorem 2.2

(Wetting regime) Let $D^\mathrm{w}_n:=\{w_1,\dots ,w_n\}\subset \partial {\mathcal {L}}_{FS}$ be any configuration such that, if $q= 1$,

30 $$\begin{aligned} w_{i+1}:=w_i+{\varvec{t}}_1\end{aligned}$$

for every $i=1,\ldots ,{n}$ and every $n\in \mathbb {N}$. It holds that $D^{\mathrm{w}}_n$ satisfies the following two assertions for every $n\in \mathbb {N}$:

(i) $$V_n(D^{\mathrm{w}}_n)=\min {V_n(D_n)}$$,
(ii) $V_n(D^{\mathrm{w}}_n)<V_n(D_n)$ for every crystalline configuration $D_n$ with $D_n{\setminus }\partial {\mathcal {L}}_{FS}\ne \emptyset$ *(*and, for the case $q= 1$, also for every configuration $D_n$ with $D_n{\setminus }\partial {\mathcal {L}}_{FS}=\emptyset$ and for which (30) does not hold*)*,

if and only if

31 $$\begin{aligned} {\left\{ \begin{array}{ll} c_{S}\ge 6c_{F} &{} \text {if }q\ne 1,\\ c_{S}\ge 4c_{F} &{} \text {if }q=1. \end{array}\right. } \end{aligned}$$

In particular, for the necessity of (31) it is enough assertion (i), and more specifically that there exists an increasing subsequence $(n_k)_{k \in {\mathbf {N}}}$ such that $V_{n_k}(D^{\mathrm{w}}_{n_k})=\min {V_{n_k}(D_{n_k})}$ holds for every $n_k$.

We refer to (31) as a *wetting condition* or as the *wetting regime*, and to the opposite condition, namely

32 $$\begin{aligned} {\left\{ \begin{array}{ll} c_{S}<6 c_{F} &{} \text {if }{q\ne 1 },\\ c_{S}< 4 c_{F} &{} \text {if }{q= 1}, \end{array}\right. } \end{aligned}$$

as the *dewetting condition* or the *dewetting regime*. The following result shows that connected components with the largest cardinality of minimizers incorporate the whole mass in the limit.

#### Theorem 2.3

(Mass conservation) Assume (32). If ${\widehat{D}}_n$ are minimizers of $V_n$ among all crystalline configurations in ${\mathcal {C}}_n$, i.e.,

$$\begin{aligned} V_n({\widehat{D}}_n)=\min _{D_n\in {\mathcal {C}}_n}{V_n(D_n)}, \end{aligned}$$

and we select for every ${\widehat{D}}_n$ a connected component ${\widehat{D}}_{n,1}\subset {\widehat{D}}_n$ with largest cardinality, then

$$\begin{aligned} \lim _{n \rightarrow \infty }{\mu _{{\widehat{D}}_n} ({\widehat{D}}_{n,1})}=1, \end{aligned}$$

where $\mu _{{\widehat{D}}_n}$ are the empirical measure associated with ${\widehat{D}}_n$ defined by (14).

We rigorously prove by $\Gamma$-convergence that the discrete models converge to the continuum model, and in view of the previous result (even in the lack of a direct compactness result for general sequences of minimizers, possibly not almost connected), we prove convergence (up to passing to a subsequence and up to translations) of the minimizers of the discrete models to a bounded minimizer of the continuum model, which in turn it is also proven to exist. We do not discuss here further the minimality property of the Winterbottom shape for the energy ${\mathcal {E}}$ and the uniqueness of the minimizers of $I_{\infty }$ in ${\mathcal {M}}_W$.

#### Theorem 2.4

(Convergence of Minimizers) Assume (32). The following statements hold:

1. The functional
  33 $$\begin{aligned} E_n:= n^{-1/2}(I_n+6c_F n), \end{aligned}$$
  where $I_n$ is defined by (16), $\Gamma$-converges with respect to the weak* convergence of measures to the functional $I_\infty$ defined by
  34 $$\begin{aligned} I_\infty (\mu ):={\left\{ \begin{array}{ll} {\mathcal {E}}(D_\mu ), &{} \text {if there exists }D_\mu \subset \mathbb {R}^2{\setminus }S\text { set of finite perimeter}\\ &{} \quad \text {with }D_\mu |=1/\rho \text { such that } \mu =\rho \chi _{D_\mu }, \\ +\infty , &{}\text {otherwise,}\\ \end{array}\right. } \end{aligned}$$
  for every $\mu \in {\mathcal {M}}(\mathbb {R}^2)$, where $\rho :=2/\sqrt{3}$.
2. The functional $I_\infty$ admits a minimizer in
  35 $$\begin{aligned} {\mathcal {M}}_W:=\bigg \{\mu \in {\mathcal {M}}(\mathbb {R}^2)\ :\ \exists D\subset \mathbb {R}^2{\setminus }S&\quad \text {set of finite perimeter, bounded }\nonumber \\ \quad \text {with } |D|=\frac{1}{\rho },&\quad \text { and such that }\mu =\rho \chi _D\bigg \}. \end{aligned}$$
3. Every sequence $\mu _n\in {\mathcal {M}}_n$ of minimizers of $E_n$ admits, up to translation in the direction ${\varvec{t}}_1$ (i.e., up to replacing $\mu _n$ with $\mu _n(\cdot +c_n{\varvec{t}}_1)$ for chosen fixed integers $c_n\in \mathbb {Z})$, a subsequence converging with respect to the weak* convergence of measures to a minimizer of $I_\infty$ in ${\mathcal {M}}_W$.

Notice that the parameter $\rho :=2/\sqrt{3}$ in the definition of ${\mathcal {M}}_W$ is related to the fact that we chose the triangular lattice for ${\mathcal {L}}_F$, as $\rho$ is the density of atoms per unit volume of such lattice.

## Wetting Regime

In this section, we single out conditions that entail *wetting*, i.e., the situation in which it is more convenient for film atoms to spread on the infinite substrate surface instead of accumulating in clusters, or islands, on top of it. In the following we refer to crystalline configurations $D^\mathrm{w}_n\subset \partial {\mathcal {L}}_{FS}$ as *wetting configurations*. We first consider the case $q\ne 1$.

### Proposition 3.1

Let $q\ne 1$ and $n \in {\mathbb {N}}$. Any wetting configuration $D^\mathrm{w}_n:=\{w_1,\dots ,w_n\}\subset \partial {\mathcal {L}}_{FS}$ satisfies the following two assertions:

(i) $$V_n(D^{\mathrm{w}}_n)=\min {V_n(D_n)}$$,
(ii) $V_n(D^{\mathrm{w}}_n)<V_n(D_n)$ for any crystalline configuration $D_n$ with $D_n{\setminus }\partial {\mathcal {L}}_{FS}\ne \emptyset$,

if and only if

36 $$\begin{aligned} c_{S} \ge 6 c_{F}. \end{aligned}$$

### Remark 3.2

Notice from the proof of Proposition 3.1 that for the necessity of (36) it is enough assertion (i) or, more precisely, it is enough that there exists an increasing subsequence $(n_k)_{k \in {\mathbf {N}}}$ such that (i) holds for every $k \in {\mathbb {N}}$. (36) is sufficient for (i) and (ii), for every $n \in {\mathbb {N}}$.

### Proof

We begin by proving the sufficiency of (36) for the assertions (i) and (ii). Note that (i) easily follows from (ii) and the fact that any wetting configuration $D^{\mathrm{w}}_n$ has the same energy given by

37 $$\begin{aligned} V_n (D^{\mathrm{w}}_n)=-c_S n. \end{aligned}$$

In order to prove (ii) we proceed by induction on *n*. We first notice that (ii) is trivial for $n=1$. Then, we assume that (ii) holds true for every $k=1,\dots ,n-1$ and prove that it holds also for *n*. Let $D_n$ be a crystalline configuration such that $D_n{\setminus }\partial {\mathcal {L}}_{FS}\ne \emptyset$. If $D_n\cap ({\mathbb {R}}\times \{r> e_{FS} \})=\emptyset$, we can easily see that the energy of $D_n$ is higher than the energy of $D^\mathrm{w}_n$ at least by $c_S-2c_F$, which is positive by (36), since the elements in $D_n{\setminus }\partial {\mathcal {L}}_{FS}\ne \emptyset$ have at most two film bonds and no substrate bonds. Therefore, we can assume that $D_n\cap ({\mathbb {R}}\times \{r> e_{FS} \})\ne \emptyset$. Let *L* be the last line in $\mathbb {R}\times \{r>0\}$ parallel to ${\varvec{t}}_1$ that intersects $D_n$ by moving upwards from $\mathbb {R}\times \{e_{FS} \}$ (which exists since $D_n$ has a finite number of atoms).

We claim that

38 $$\begin{aligned} V_n(D_n) \ge V_{n-\ell }(D_n\backslash L)-6c_F(\ell -1)-4c_F, \end{aligned}$$

where $\ell :=\#(D_n \cap L)$. We order the elements of $D_n \cap L$ with increasing indexes with respect to ${\varvec{t}}_1$, i.e., $D_n \cap L=\{x_1,\dots ,x_\ell \}$, and observe that $x_1$ has at most 3 bonds with film atoms in $D_n$ by construction, since $x_1$ is the leftmost element in $D_n \cap L$. We notice that in the same way, if $\ell >1$, every $x_i$ has at most 3 bonds with film atoms in $D_n{\setminus }\{x_1,\dots ,x_{i-1}\}$ for every $i=2,\dots ,\ell -1$. Therefore, we obtain that

$$\begin{aligned} V_n(D_n)&\ge V_{n-1}(D_{n-1} \backslash \{x_1\})-6c_F\ge V_{n-i}(D_{n-i}\backslash \{x_1,\dots ,x_{i}\})-6c_Fi\\&\ge V_{n-(\ell -1)}(D_n\backslash \{x_1,\dots ,x_{\ell -1}\})-6c_F(\ell -1) \\&\ge V_{n-\ell }(D_n\backslash L)-6c_F(\ell -1)-4c_F, \end{aligned}$$

which in turns is (38), where in the last inequality we used that $x_\ell$ has only at most 2 bonds with film atoms in $D_n{\setminus }L$, since $x_\ell$ is the rightmost element in $D_n \cap L$.

From (38), it follows that

$$\begin{aligned} V_n(D_n)&\ge V_{n-\ell }(D_n\backslash L)-6c_F(\ell -1)-4c_F> V_{n-\ell }(D_n\backslash L)-6c_F\ell \\&\ge -c_S (n-\ell )-6c_F\ell \ge -c_Sn, \end{aligned}$$

where we used the induction and (37) in the third inequality, and (36) in the last inequality.

To prove the necessity of (36), notice that the Wulff configuration in $\mathbb {R}\times \{r> e_{FS} \}$ has energy equal to $-6c_Fn+C\sqrt{n}$ for some constant $C>0$. Therefore, from assertion (ii) and (37) it follows

$$\begin{aligned} -c_S n < -6c_Fn +C \sqrt{n}. \end{aligned}$$

After dividing by *n* and letting $n \rightarrow \infty$ we obtain $c_S \ge 6 c_F$. $\square$

We now address the case $q=1$ for which we notice that $\partial {\mathcal {L}}_{FS}=\partial {\mathcal {L}}_{F}$.

### Proposition 3.3

Let $q= 1$ and $n \in {\mathbb {N}}$. Any configuration $D^\mathrm{w}_n:=\{w_1,\dots ,w_n\}\subset \partial {\mathcal {L}}_{FS}$ such that

39 $$\begin{aligned} w_{i+1}:=w_i+{\varvec{t}}_1\end{aligned}$$

for every $i=1,\dots ,{n}$, satisfies the following two assertions:

(i) $$V_n(D^{\mathrm{w}}_n)=\min {V_n(D_n)}$$,
(ii) $V_n(D^{\mathrm{w}}_n)<V_n(D_n)$ for any crystalline configuration $D_n$ such that either $D_n{\setminus }\partial {\mathcal {L}}_{FS}\ne \emptyset$ or not satisfying (39),

if and only if

40 $$\begin{aligned} c_{S}\ge 4 c_{F}. \end{aligned}$$

### Proof

The proof is based on the same arguments employed for Proposition 3.1 and on the following observations. Any wetting configuration $D^{\mathrm{w}}_n$ satisfying (39) has the same energy given by

41 $$\begin{aligned} V_n (D^{\mathrm{w}}_n)=-c_S n -2c_F(n-1). \end{aligned}$$

In order to prove the sufficiency of (40) for assertion (ii) (assertion (i) follows in view of (41)), we can restrict also in this case without loss of generality to configurations $D_n\cap ({\mathbb {R}}\times \{r>e_{FS} \})\ne \emptyset$, since any wetting configuration that does not satisfy (39) has energy obviously higher than (41) (because $n-1$ is the maximum number of bonds in $\partial {\mathcal {L}}_{F}$).

In order to prove the necessity of (40) for assertions (i) and (ii), we again consider the Wulff shape with *n* atoms in $\mathbb {R}\times \{r>e_{FS} \}$ which has energy $-6c_Fn+C\sqrt{n}$ for some constant $C>0$, and observe that

$$\begin{aligned} -c_S n -2c_F(n-1) < -6c_Fn +C \sqrt{n} \end{aligned}$$

by assertion (ii) and (41). $\square$

We refer to (36) and (40) as *wetting conditions*. Condition (40) is weaker than (36) because if $q=1$, then film atoms of wetting configurations can be bonded to the two film atoms at their sides in $\partial {\mathcal {L}}_{FS}$ (if filled) besides to their corresponding substrate atom, and Proposition 3.3 shows that such configuration are preferable.

### Proof of Theorem 2.2

The assertion directly follows from Propositions 3.1 and 3.3 for the case $q\ne 1$ and the case $q=1$, respectively. $\square$

## Compactness

In the remaining part of the paper, we work in the dewetting regime, i.e., under the assumption (32). We begin by establishing a lower bound in terms of $c_F$ and $c_S$ of the strip energy $E_{\mathrm{strip}}(x)$ uniform for every $x\in D_n\cap \partial {\mathcal {L}}_F$. To this aim, we need to distinguish the case $q= 1$ from $q\ne 1$ as already done in Sect. 3 because of different contributions in $E_{\mathrm{strip}}(x)$ of the substrate interactions.

### Lemma 4.1

We have that

$$\begin{aligned} E_{\mathrm{strip}}(x)\ge \Delta _{\mathrm{strip}} \end{aligned}$$

with

42 $$\begin{aligned} \Delta _{\mathrm{strip}}:={\left\{ \begin{array}{ll} 6c_F-c_S, &{}\text {if } q\ne 1 ,\\ 4c_F-c_S, &{}\text {if } q= 1 , \end{array}\right. } \end{aligned}$$

for every $$x\in D_n\cap \partial {\mathcal {L}}_F$$.

### Proof

Fix $x\in D_n\cap \partial {\mathcal {L}}_F$. We begin by observing that the strip center *x* surely misses the bonds with the atoms missing at the 2 positions $x-{\varvec{t}}_2+k{\varvec{t}}_1$ for $k=0,1$ as shown in Fig. 3. Furthermore, either *x* misses the bond with $x_{-}$ or $x_{-}\in {D_n}$ and $x_{-}$ misses the bonds with the 2 positions $x-{\varvec{t}}_2+k{\varvec{t}}_1$ for $k=-1,0$ (which in the strip energy are counted with half weights). We can reason similarly for $x_{+}$. Therefore, by the definition of energy of the low strip $E_\mathrm{strip,below}$,

$$\begin{aligned} E_{\mathrm{strip,below}}\ge {\left\{ \begin{array}{ll} 4c_F-c_S, &{}\text {if }q\ne 1,\\ 2c_F-c_S, &{}\text {if }q=1, \end{array}\right. } \end{aligned}$$

We analyze $E_{strip, above}$. There are several possibilities:

1. neither of ${\tilde{x}}_+$ and ${\tilde{x}}_-$ belongs to $D_n$;
2. exactly one of ${\tilde{x}}_+$ and ${\tilde{x}}_-$ belongs to $D_n$;
3. both ${\tilde{x}}_+$ and ${\tilde{x}}_-$ belong to $D_n$.

In case of (1) we have the contribution of $2c_F$ since ${{\tilde{x}}}$ misses two bonds (in the case when ${{\tilde{x}}}=x$ this contribution comes additionally in $E_{\mathrm{loc}}(x)$, i.e., $E_\mathrm{strip,below}$). In case of (3) each of ${\tilde{x}}_+$ and ${\tilde{x}}_-$ misses at least one bond (namely with ${\tilde{x}}+2{\varvec{t}}_2-{\varvec{t}}_1$ which is not in $D_n$ due to the definition of ${{\tilde{x}}}$). If ${\tilde{x}}_{\pm }\ne \widetilde{(x_{\pm })}_{\mp }$ we have the energy contribution of at least $2c_F$. On the other hand if it is valid that ${\tilde{x}}_{\pm }= \widetilde{(x_{\pm })}_{\mp }$, we have the energy contribution of $c_F$ due to the missing bond with ${\tilde{x}}+2{\varvec{t}}_2-{\varvec{t}}_1$ and each of ${\tilde{x}}_{\pm }$ misses one more bond (namely with ${\tilde{x}}+2{\varvec{t}}_2$ and ${\tilde{x}}+2{\varvec{t}}_2-2{\varvec{t}}_1$, which in this case do not belong to $D_n$). The similar analysis can be made if ${\tilde{x}}_{+}= \widetilde{(x_{+})}_{-}$ or ${\tilde{x}}_{-}= \widetilde{(x_{-})}_{+}$. Thus, we have again energy deficiency of $2c_F$. Finally in the case of (2) without loss of generality we assume that ${{\tilde{x}}}_{+} \in D_n$. ${{\tilde{x}}}$ is already missing one bond (one $c_F$), which is again in the case ${{\tilde{x}}}=x$ counted in $E_{\mathrm{loc}}(x)$, i.e., $E_{\mathrm{strip, below}}$. And again one bond of ${{\tilde{x}}}_{+}$ is missing since ${\tilde{x}}+2{\varvec{t}}_2-{\varvec{t}}_1$ is not in $D_n$. Again, this bond is counted as one $c_F$, if ${\tilde{x}}_{+}\ne \widetilde{(x_{+})}_{-}$ and as $c_F/2$, if ${\tilde{x}}_{+}=\widetilde{(x_{+})}_{-}$. In this case one more $c_F/2$ we obtain since ${\tilde{x}}_{+}$ is missing one bond with ${\tilde{x}}+2{\varvec{t}}_2$.

Therefore, in the strip energy $E_{\mathrm{strip}}$ the terms related to the triple ${\tilde{x}}$, ${\tilde{x}}_+$, and ${\tilde{x}}_-$ give a contribution of at least $2c_F$. $\square$

We now observe that the energy $V_n(D_n)$ of any crystalline configuration $D_n$ is bounded below by $-6c_Fn$ plus a positive *deficit* due to the boundary of $D_n$ where atoms have less than 6 film bonds and could have a bond with the substrate.

### Lemma 4.2

If (32) holds, then there exists $\Delta >0$ such that

43 $$\begin{aligned} V_n(D_n)\ge -6c_Fn\,+\,\Delta \#\partial D_n \end{aligned}$$

for every crystalline configuration $D_n\subset {\mathcal {L}}_F$. Furthermore, the following two assertions are equivalent:

(i) There exists a constant $C>0$ such that $\# \partial D_n \le C \sqrt{n}$ for every $n \in {\mathbb {N}}$,
(ii) There exists a constant $C'>0$ such that $E_n (\mu _{D_n}) \le C'$ for every $n \in {\mathbb {N}}$.

### Proof

We begin by observing that from (18) and (21) it follows that

44 $$\begin{aligned} 6c_Fn\,+\, V_n(D_n)\,&=\, \sum _{x\in D_n}\left( \sum _{y\in {D_n}{\setminus }\{x\}} v_{FF}(|x-y|)+ 6c_F \right) \,+\,\sum _{x\in D_n} v^1(x)\nonumber \\&=\, \sum _{x\in D_n} E_{\mathrm{loc}}(x)\,+\,\sum _{x\in D_n} v^1(x)\nonumber \\&\ge \, \sum _{x\in D_n\cap {\partial {\mathcal {L}}_{FS}}} E_\mathrm{strip}(x)\,+\,\sum _{x\in D_n{\setminus }{\mathcal {S}}({\partial {\mathcal {L}}_{FS}})} E_{\mathrm{loc}}(x), \end{aligned}$$

where

45 $$\begin{aligned} {\mathcal {S}}(\partial {\mathcal {L}}_{FS})={\mathcal {S}}_{ D_n}({\partial {\mathcal {L}}_{FS}}):=\{y\in {\mathcal {S}}(x)\,:\,x\in D_n\cap {\partial {\mathcal {L}}_{FS}} \}, \end{aligned}$$

because $v^1(x)=0$ for every $x\in D_n{\setminus }\partial {\mathcal {L}}_{FS}$ and the careful choice of the weights in (22), (23), and (24) with (25). More precisely, we notice that for every point in $D_n\cap {\partial {\mathcal {L}}_{FS}}$ the local energy $E_{\mathrm{loc}}(x)$ is counted at most once. The weights $w_{\pm }({\tilde{x}}_{\pm })$are instead chosen so that the local energy of ${\tilde{x}}_{\pm }$ is fully counted if ${\tilde{x}}_{\pm }$ do not belong to the next strip and only half in the other case. Thus, these weights are also at most one. We now observe that

46 $$\begin{aligned} \sum _{x\in D_n{\setminus }{\mathcal {S}}(\partial {\mathcal {L}}_{FS})} E_{\mathrm{loc}}(x)\ge c_F \#(\partial D_n{\setminus }{\mathcal {S}}({\partial {\mathcal {L}}_{FS}})) \end{aligned}$$

because $E_{\mathrm{loc}}(x)=0$ for every point $x\in D_n{\setminus }{\mathcal {S}}(\partial {\mathcal {L}}_{FS})$ that does not belong to $\partial D_n$ where at least one bond is missing by definition.

Therefore, by (44), (46), and Lemma 4.1 we obtain that

47 $$\begin{aligned} 6c_F n\,+\, V_n(D_n)\,&\ge \, \sum _{x\in D_n\cap {\partial {\mathcal {L}}_{FS}}} E_{\mathrm{strip}}(x)\,+\,\sum _{x\in D_n{\setminus }{\mathcal {S}}({\partial {\mathcal {L}}_{FS}} )} E_\mathrm{loc}(x),\nonumber \\&\ge \Delta _{{\mathrm{strip}}} \#(D_n\cap {\partial {\mathcal {L}}_{FS}}) \,+\, c_F \#(\partial D_n{\setminus }{\mathcal {S}}({\partial {\mathcal {L}}_{FS}}))\nonumber \\&\ge \min \left\{ \frac{\Delta _{{\mathrm{strip}}}}{6}, c_F\right\} \#\partial D_n \end{aligned}$$

where in the last inequality we used that $\#{\mathcal {S}}(\partial {\mathcal {L}}_{FS})\le 6\#D_n \cap \partial {\mathcal {L}}_{FS}$. The assertion now easily follows from (47) by choosing

$$\begin{aligned} \Delta :=\min \left\{ \frac{\Delta _{{\mathrm{strip}}}}{6}, c_F\right\} >0, \end{aligned}$$

where we used (32).

To prove the last assertion, we observe that assertion (i) implies (ii) since by (17) and (33)

$$\begin{aligned} \sqrt{n}E_n(\mu _{D_n})=V_n(D_n)+6c_Fn\le 6c_F\#\partial D_n, \end{aligned}$$

where in the last equality we used the definition of $\partial D_n$. Furthermore, also by (43),

$$\begin{aligned} \Delta \#\partial D_n\le V_n(D_n)+6c_Fn=\sqrt{n}E_n(\mu _{D_n}) \end{aligned}$$

and hence, assertion (ii) implies (i). $\square$

In view of the previous lower bound for the energy of a configuration $D_n$, we are now able to prove a compactness results. We notice that to achieve compactness the negative contribution coming at the boundary from the interaction with the substrate needs to be compensated. This is not trivial, e.g., in the case $6c_F>c_S>4c_F$, where atoms *x* of configurations on $\partial {\mathcal {L}}_{FS}$ have one bond with a substrate atom and at least two bonds with film atoms missing. A way to solve the issue is to look for extra positive contributions from other atoms in the boundary. However, just looking for neighboring atoms might be not enough, e.g., in the case with $e_{FS}=e_S=2$ or $e_{FS}=e_S=\frac{2}{3}$. The issue is solved in the proof of the following compactness result by introducing a new non-local argument called the *strip argument* that involve looking at the whole strip ${\mathcal {S}}(x)$.

We conclude the section with compactness results for sequences of almost-connected configuration (see Sect. 2.4 for the definition). We remind the reader that by the transformation defined in Definition 2.1 for any configuration $D_n$ there exists the almost-connected configuration ${\widetilde{D}}_n$ such that $V_n({\widetilde{D}}_n)\le V_n(D_n)$.

### Proposition 4.3

Assume that (32) holds. Let $D_n\in {\mathcal {C}}_n$ be almost-connected configurations such that

48 $$\begin{aligned} V_n(D_n)\le -6c_Fn +Cn^{1/2} \end{aligned}$$

for a constant $C>0$. Then, there exist an increasing sequence $n_r$, $r\in \mathbb {N}$, and a measure $\mu \in {\mathcal {M}}(\mathbb {R}^2)$ with $\mu \ge 0$ and $\mu (\mathbb {R}^2)=1$ such that $\mu _{r}{\mathop {\rightharpoonup }\limits ^{*}} \mu$ in ${\mathcal {M}}(\mathbb {R}^2)$, where $\mu _{r}:=\mu _{D_{n_r}(\,\cdot \,+a_{n_r})}$ for some translations $a_n\in \mathbb {R}^2$ *(*see 14 for the definition of the empirical measures $\mu _{D_{n_r}}$*)*. Moreover, if $D_n\in {\mathcal {C}}_n$ are minimizers of $V_n$ in ${\mathcal {C}}_n$, then we can choose $a_n=t_n{\varvec{t}}_1$ for integers $t_n\in \mathbb {Z}$.

### Proof

We follow the approach of Au Yeung et al. (2012, Proposition 3.2) with the necessary modification to include almost-connected configurations, which are not necessarily connected. In the following we denote by *B*(*x*, *R*) an open ball of radius $R>0$ centered at $x \in {\mathbb {R}}^2$ and we define $B(R):=B(o,R)$ where *o* is the origin in ${\mathbb {R}}^2$. We want to show that there exists $R>0$ such that $D_{n}\subset B(R)$ (up to a translation) for every *n*.

To this aim we denote for any $D_n$ its $k:=k_{D_n}$ connected components by $D_n^i$ for $i=1,\dots ,k$. We define the sets

$$\begin{aligned} \Omega _i:= \bigcup _{x\in D_n^i} \nu _{\text {trunc}}(x), \end{aligned}$$

for $$i=1,\dots ,k$$, where

$$\begin{aligned} \nu _{\text {trunc}}(x):=\nu (x)\cap B(x,q) \end{aligned}$$

with *q* defined in (13) and $\nu (x)$ denoting the (closed) Voronoi cell associated with *x* with respect to $D_n^{i}$, i.e.,

49 $$\begin{aligned} \nu (x):= \{ y\in \mathbb {R}^2\ :\ |y-x|\le |y-x'|\text { for all }x'\in D_n^{i}{\setminus }\{x\}\}, \end{aligned}$$

and we observe that by construction and the convexity of $\nu (x)$,

50 $$\begin{aligned} |\partial \nu _{\text {trunc}}(x)|\le 2 q \pi . \end{aligned}$$

We claim that $\Omega _i$ are connected. Indeed, if $x,y \in D_n$ are such that $|x-y|=1$, then it is easily seen that the midpoint on the line that connects *x* and *y* belongs to both $\nu (x) \cap \nu (y)$ and $B (x,q) \cap B (y,q)$. The second inclusion easily follows from (13) while the first inclusion follows from the triangular inequality (it is impossible that for some $z \in D_n$ it is valid

$$\begin{aligned} \left| z- \frac{x+y}{2}\right| <1/2 \end{aligned}$$

since then by the triangular inequality *z* would be distant from both *x* and *y* less than one).

We now claim that also

$$\begin{aligned} \Omega := \bigcup _{x\in D_{n}} \nu _{\text {trunc}}(x), \end{aligned}$$

is connected. This follows by showing that $\Omega _i$ and $\cup _{i=1}^{i-1}\Omega _l$ are connected for $i=2, \dots , k$, which in turns is a consequence of the fact that by definition $D_n^i$ is separated by at most *q* from $\cup _{l=1}^{i-1} D_n^l$ for $i=2,\dots ,k$. In fact, by the same reasoning used in the previous claim applied this time to two points $x \in D_n^i$ and $y \in \cup _{l=1}^{i-1}D_n^l$ chosen such that $|x-y|= \text {dist}(D_n^i,\cup _{l=1}^{i-1} D_n^l)$, where $\text {dist}(A,B)$ with respect to two subsets *A* and *B* of ${\mathbb {R}}^2$ denotes the distance between them, we can deduce that $(x+y)/2$ belongs to both $\nu (x) \cap \nu (y)$ and $B(x,q) \cap B (y,q)$, which yields the claim. Since the interior of the set $\Omega$, denoted by $\mathring{\Omega }$ is open, connected, of finite measure, and satisfies $\mathring{\Omega }^1=\mathring{\Omega }$, where $\mathring{\Omega }^1$ denotes the set of points in $\mathbb {R}^2$ of density one for $\mathring{\Omega }$ (Ambrosio et al. 2000), we have that (see Dayrens et al. 2019, Remark 2.2 and Lemma 2.13)

$$\begin{aligned} \text {diam}(\Omega )\le \frac{1}{2} |\partial \Omega |. \end{aligned}$$

Therefore, we have that

51 $$\begin{aligned} \text {diam}(D_{n}):=\max _{x,y\in D_{n}} |x-y|\le \text {diam}(\Omega )\le \frac{1}{2} |\partial \Omega |\le \frac{1}{2} \sum _{x\in \partial D_n}|\partial \nu _{\text {trunc}}(x)| \le \pi q \#\partial D_{n} \end{aligned}$$

where $\text {diam} (A)$ of a set *A* is the diameter of *A* and we used that $\Omega$ is connected in the second inequality, that if $x \in D_n$ has 6 film neighbors, then by elementary geometric observations $\nu _{\text {trunc}} (x) \cap \partial \Omega =\emptyset$ in the third inequality, and (50) in the last inequality.

Finally, from (48), (51) and Lemma 4.2 we obtain that

$$\begin{aligned} \text {diam}(D_{n})\le \frac{C\pi q}{\Delta } n^{1/2} \end{aligned}$$

and hence, by (14) there exist translations $\mu _n$ of $\mu _{D_n}$ such that $\text {supp} \mu _n\subset B(R)$ for some $R>C\pi q/2\Delta$ and for every *n*. Therefore, since $|\mu _{D_n}|(\mathbb {R}^2)=1$ for every *n*, by Ambrosio et al. (2000, Theorem 1.59) there exist a subsequence $(n_r)_{r} \in {\mathbf {N}}$ and a measure $\mu \in {\mathcal {M}}(\mathbb {R}^2)$ such that $\mu _{r}{\mathop {\rightharpoonup }\limits ^{*}}\mu$ in ${\mathcal {M}}(\mathbb {R}^2)$. Furthermore, $\mu \ge 0$ and

$$\begin{aligned} \mu (\mathbb {R}^2)\le \lim _{r\rightarrow \infty } {\mu }_r(\mathbb {R}^2)=1. \end{aligned}$$

In order to conclude the proof it suffices to prove that $\mu (\mathbb {R}^2)=1$, and this directly follows from the fact that the support of $\mu _r$ are contained in a compact set of $\mathbb {R}^2$. The last claim follows from the fact that if $D_n \in {\mathcal {C}}_n$ is a minimizer of $V_n$ then we have that ${\mathcal {T}}_1 (D_n)=D_n$, i.e., all connected components of $D_n$ are connected with the substrate and $D_n$ is almost-connected. $\square$

The following compactness result is the analogous of Au Yeung et al. (2012, Theorem 1.1) in our setting with substrate interactions.

### Theorem 4.4

(Compactness) Assume (32). Let $D_n\in {\mathcal {C}}_n$ be configurations satisfying (48) and let $\mu _{n}:=\mu _{{\mathcal {T}}(D_n)}$ be the empirical measures associated with the transformed configurations ${\mathcal {T}}(D_n)\in {\mathcal {C}}_n$ associated with $D_n$ by Definition 2.1. Then, up to translations (i.e., up to replacing $\mu _{n}$ by $\mu _{n}(\cdot +a_n)$ for some $a_n\in \mathbb {R}^2 )$ and a passage to a non-relabeled subsequence, $\mu _{n}$ converges weakly * in ${\mathcal {M}}({\mathbb {R}}^2)$ to a measure $\mu \in {\mathcal {M}}_W$, where ${\mathcal {M}}_W$ is defined in (35). Furthermore, if $D_n\in {\mathcal {C}}_n$ are minimizers of $V_n$ in ${\mathcal {C}}_n$, then we can choose $a_n=t_n{\varvec{t}}_1$ for integers $t_n\in \mathbb {Z}$.

### Proof

We begin by observing that the transformed configurations ${\mathcal {T}}(D_n)$ of the configurations $D_n$ are almost-connected configurations in ${\mathcal {C}}_n$ since they result from applying transformation ${\mathcal {T}}_2$, and that

52 $$\begin{aligned} V_n({\mathcal {T}}(D_n))\le V_n(D_n), \end{aligned}$$

since no active bond of $D_n$ is deactivated by performing the transformations ${\mathcal {T}}_1$ and ${\mathcal {T}}_2$ (see Definition 2.1 for the definition of ${\mathcal {T}}_1$ and ${\mathcal {T}}_2$). Therefore, in view of Proposition 4.3 by (48) and (52) we obtain that, up to a non-relabeled subsequence, there exist $a_n\in \mathbb {R}^2$ and a measure $\mu \in {\mathcal {M}}(\mathbb {R}^2)$ with $\mu \ge 0$ and $\mu (\mathbb {R}^2)=1$ such that

$$\begin{aligned}\mu _{{\mathcal {T}}(D_n)}(\cdot +a_n)\rightharpoonup ^*\mu \end{aligned}$$

in ${\mathcal {M}}(\mathbb {R}^2).$ We can then conclude that $\mu \in {\mathcal {M}}_W$ by directly applying the arguments in the proof of Au Yeung et al. (2012, Theorem 1.1). $\square$

We notice that, if the sequence $D_n\in {\mathcal {C}}_n$ is a sequence of almost-connected configurations, then Theorem 4.4 directly holds for $D_n$ without the need to pass to the associated transformed configurations ${\mathcal {T}}(D_n)$ given by Definition 2.1.

## Lower Bound

We denote by $h_{1/\sqrt{3}}(x)$ the interior part of the Voronoi cell associated with every $x\in {\mathcal {L}}_F$ with respect to ${\mathcal {L}}_F$, i.e.,

$$\begin{aligned} h_{1/\sqrt{3}}(x):= \left\{ y\in \mathbb {R}^2\ :\ |y-x|<|y-x'|\text { for all }x'\in {\mathcal {L}}_F{\setminus }\{x\}\right\} \end{aligned}$$

that is an open hexagon of radius $1/\sqrt{3}$, and by *v*(*x*) its scaling in ${\mathcal {L}}_F/\sqrt{n}$, i.e.

53 $$\begin{aligned} v(x):= \frac{h_{1/\sqrt{3}}(x)}{\sqrt{n}}. \end{aligned}$$

Given a configuration $D_n$, we consider the auxiliary set $H_n$ associated with $D_n$ which was introduced in Au Yeung et al. (2012) and defined by

54 $$\begin{aligned} H_n= \bigcup _{x \in D_n} \overline{v(x)}. \end{aligned}$$

**Fig. 4:**
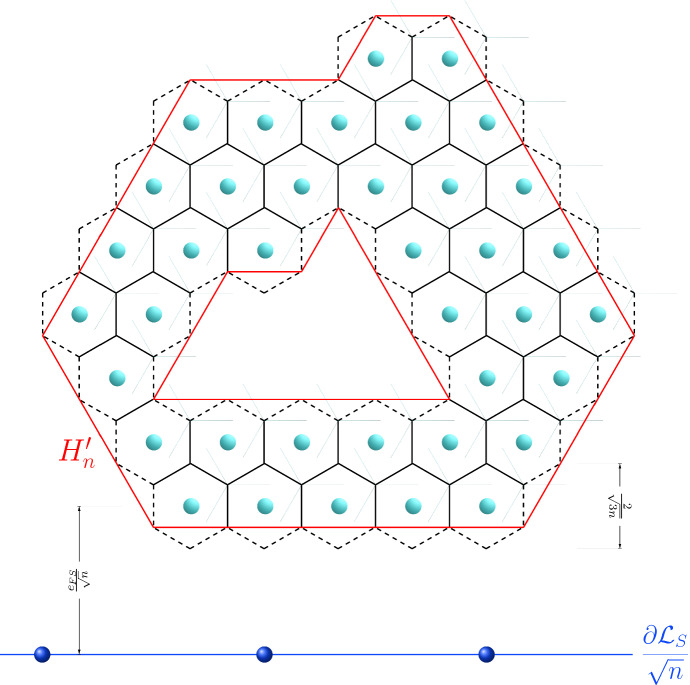
A configuration $D_n/\sqrt{n}$ is depicted with scaled Voronoi cells *v*(*x*) for every $x\in D_n$. The boundary of $H_n$, which in this example consists of two polygonal lines (one “internal” and one “external”), is indicated with a dashed black line while the boundary of $H_n'$ with a continuous red line

The boundary of $H_n$ is given by the union of a number $M\in \mathbb {N}$ (depending on $D_n$) of closed polygonal boundaries $P_1,\dots ,P_M$. For $k=1,\dots ,M$ we denote the $m_k\in \mathbb {N}$ vertices of $P_k$ by $v_1^k,\dots ,v_{m_k}^k$ and we set $v_{m_k+1}^k:=v_1^k$, so that

$$\begin{aligned} P_k:=\bigcup _{i=1}^{m_k}[v_{i+1}^k,v_i^k] \end{aligned}$$

where [*a*, *b*] denotes the closed segment with endpoints $a,b\in \mathbb {R}^2$. Notice that each $m_k$ is even and that we can always order the vertices so that

$$\begin{aligned} v_{2i}^k\in V_{{\mathcal {L}}_F}^{\mathrm{e}}:=\left( \frac{1}{3\sqrt{n}}({\varvec{t}}_1+{\varvec{t}}_2)+\frac{1}{\sqrt{n}}{\mathcal {L}}_F\right) \end{aligned}$$

and

$$\begin{aligned} v_{2i-1}^k\in V_{{\mathcal {L}}_F}^\mathrm{o}:=\left( \frac{1}{3\sqrt{n}}(2{\varvec{t}}_1-{\varvec{t}}_2)+\frac{1}{\sqrt{n}}{\mathcal {L}}_F\right) \end{aligned}$$

(see Fig. 4). To avoid the atomic-scale oscillations in $\partial H_n$ between the two sets of vertices $V_{{\mathcal {L}}_F}^{\mathrm{e}}$ and $V_{{\mathcal {L}}_F}^{\mathrm{o}}$, we introduce another auxiliary set denoted by $H'_n$ where such oscillations are removed, by considering only the vertices in one of the two sets, say $V_{{\mathcal {L}}_F}^{\mathrm{o}}$ as depicted in Fig. 4. More precisely, the set $H'_n\subset \mathbb {R}^2$ is defined as the unique set with $D_n\subset H_n'$ such that

55 $$\begin{aligned} \partial H_n':=\bigcup _{k=1}^M P_k', \end{aligned}$$

where

$$\begin{aligned} P_k':=\bigcup _{i=1}^{m_k/2}[v_{2i-1}^k,v_{2i+1}^k]. \end{aligned}$$

It easily follows from the construction of the auxiliary sets $H_n$ and $H_n'$ associated with the configuration $D_n$ that

56 $$\begin{aligned} |H_n \Delta H_n'| \le \frac{\# \partial D_n}{8n \sqrt{3}}, \end{aligned}$$

and

57 $$\begin{aligned} \left| {\mathcal {H}}^1(\partial H_n)-{\mathcal {H}}^1(\partial H_n')\right| \le 2\sqrt{3} \frac{\# \partial D_n}{\sqrt{n}}. \end{aligned}$$

In the following we use the notation

$$\begin{aligned} \partial {\mathcal {L}}_{FS}^n:= \frac{\partial {\mathcal {L}}_{FS}}{\sqrt{n}}, \quad \partial {\mathcal {L}}_{F}^n:= \frac{\partial {\mathcal {L}}_{F}}{\sqrt{n}}. \end{aligned}$$

For every point $y\in \partial {\mathcal {L}}_{F}^n$ we denote its left and right half-open intervals with length $1/(2\sqrt{n})$ by

$$\begin{aligned} I^{+}_y:=\left[ y,y+\frac{1}{2\sqrt{n}}\right) \quad \text {and}\quad I^{-}_y:=\left( y-\frac{1}{2\sqrt{n}}, y\right] , \end{aligned}$$

respectively, and we associate with $y\in \partial {\mathcal {L}}_{F}^n$ the set

$$\begin{aligned} O_n^{y}:=O_n^{y,-}\cup O_n^{y,+}, \end{aligned}$$

where $O_n^{y,\pm }$, for $y\in \partial {\mathcal {L}}_{F}^n$ is

$$\begin{aligned} O_n^{y,\pm }:=\{I^{\pm }_y\times \mathbb {R}: y\in \partial {\mathcal {L}}_{F}^n\}. \end{aligned}$$

The *oscillatory set* $O_n$ (see Fig. 2) is defined as

58 $$\begin{aligned} O_n:=\bigcup _{y\in \partial {\mathcal {L}}_{FS}^n}O_n^{y}. \end{aligned}$$

Here $O_n$ is the oscillatory set that consists of union of stripes of width $1/\sqrt{n}$ and infinite length that correspond to the possible positions of film atoms at the place $x_2=\frac{e_{FS} }{\sqrt{n}}$ that are at distance $\frac{e_{FS} }{\sqrt{n}}$ from some of substrate atoms. $\nu _{H_n'}$ is a normal at the boundary.

The following lemma will help in the proof of the lower-semicontinuity result. It is a simplified version of the proof of Au Yeung et al. (2012, Theorem 1.1) and we give it for the sake of completeness. We recall that $\rho :=2/\sqrt{3}$.

### Lemma 5.1

Let $D_n \in {\mathcal {C}}_n$ be such that $E_n(\mu _{D_n})$ is bounded, where $\mu _{D_n}$ is the empirical measure associated with $D_n$. Let $H_n'$ be defined as above. Then, we have that $\mu _{D_n}- \rho \chi _{H_n'} {\mathop {\rightharpoonup }\limits ^{*}} 0$.

### Proof

It is easy to see that

$$\begin{aligned} \mu _{D_n}- \rho \chi _{H_n} {\mathop {\rightharpoonup }\limits ^{*}} 0. \end{aligned}$$

Namely, for $\psi \in C_0({\mathbb {R}}^2)$, where $C_0(\mathbb {R}^2)$ denotes the set of continuous functions with compact support in $\mathbb {R}^2$, we have that

$$\begin{aligned} \left| \int _{{\mathbb {R}}^2}\psi d \mu _{D_n}-\int _{{\mathbb {R}}^2}\rho \chi _{H_n}\psi dx\right|\le & {} \frac{1}{n} \sum _{x \in D_n}\sup _{x \in D_n} \left\{ |\psi (x)-\psi (y)|: |x-y|\le \frac{1}{\sqrt{3n}}\right\} \\\rightarrow & {} 0, \end{aligned}$$

as $n \rightarrow \infty$. From estimate (56) and Lemma 4.2 we have that $\rho \chi _{H_n}-\rho \chi _{H_n'} \rightarrow 0$ strongly in $L^1$, from which we have the claim. $\square$

Before proving the lower-semicontinuity result we give one simple lemma.

### Lemma 5.2

Let $\Omega \subset {\mathbb {R}}^d$ be an open set. Let $\kappa _n$ and $\mu _n$ be two sequences of finite (positive) Borel measures on the $\sigma$-algebra on $\Omega$ denoted by ${\mathcal {B}} (\Omega )$ such that:

(i) $$\sup _{n \in {\mathbb {N}} } (\mu _n(\Omega )+\kappa _n(\Omega ))< \infty $$,
(ii) $(\kappa _n)_n$ is uniformly absolutely continuous with respect to $(\mu _n)_n$, i.e., for every $\varepsilon >0$ there exists $\delta >0$ such that
  $$\begin{aligned} \mu _n(A)< \delta \implies \kappa _n (A) < \varepsilon , \end{aligned}$$
  for every $A \in {\mathcal {B}} (\Omega )$ and $n \in {\mathbb {N}}$.

If there exist Borel measures $\kappa$ and $\mu$ on ${\mathcal {B}} (\Omega )$ such that $\kappa _n {\mathop {\rightharpoonup }\limits ^{*}} \kappa$ and $\mu _n {\mathop {\rightharpoonup }\limits ^{*}} \mu$, then $\kappa$ is absolutely continuous with respect to $\mu$.

### Proof

Take $A \subset \Omega$ such that $\mu (A)=0$. Since $\kappa$ is a regular Borel measure it is enough to prove that $\kappa (K)=0$ for every $K \subset A$, *K* compact. Take an arbitrary $K \subset A$ compact and $\varepsilon >0$. By regularity of $\mu$ there exists $U \subset X$ open such that $A \subset U$ and $\mu (U) < \delta$, where $\delta$ is given by (ii). For every $x \in K$ we find a ball of radius $r_x$ such that $\mu (\partial B(x,r_x))=0$ and $B(x,r_x) \subset U$. Since *K* is compact we can find a finite number of balls $(B(x_i, r_{x_i}))_{i=1,\dots , n}$ that cover *K* and we define an open set $V \subset U$ as $V:= \cup _{i=1}^n B(x_i, r_{x_i})$. Obviously $\mu (\partial V)=0$. We have that $\mu (V) < \delta$ and $\mu _n(V) \rightarrow \mu (V)$. Thus, there exists $n_0 \in {\mathbb {N}}$ such that $\mu _n(V) < \delta$, $\forall n \ge n_0$. But then we have that $\kappa _n(V)<\varepsilon$, $\forall n \ge n_0$. By the definition of weak star convergence we also have that

$$\begin{aligned} \kappa (K) \le \kappa (V) \le \liminf _{n \rightarrow \infty } \kappa _n(V) < \varepsilon . \end{aligned}$$

The claim follows by the arbitrariness of $\varepsilon$. $\square$

The following lower-semicontinuity result for the discrete energies $E_n$ is based on adapting some ideas used in Alberti and De Simone (2005) and Fonseca and Müller (1993).

### Theorem 5.3

If $\{D_n\}$ is a sequence of configurations such that

$$\begin{aligned} \mu _{D_n} {\mathop {\rightharpoonup }\limits ^{*}} \rho \chi _{D} \end{aligned}$$

weakly* with respect to the convergence of measures, where $\mu _{D_n}$ are the associated empirical measures of $D_n$ and $D\subset \mathbb {R}^2{\setminus }S$ is a set of finite perimeter with $|D|=1/\rho$, then

59 $$\begin{aligned} \liminf _{n \rightarrow \infty } E_n (\mu _{D_n})\ge & {} {\mathcal {E}}(D). \end{aligned}$$

### Proof

Let $\{D_n\}\subset {\mathcal {C}}_n$ be a sequence of configurations such that $\mu _{D_n} {\mathop {\rightharpoonup }\limits ^{*}} \rho \chi _D$ weakly* with respect to the convergence of measures, for a set $D\subset \mathbb {R}^2{\setminus }S$ of finite perimeter with $|D|=\sqrt{3}/2$. We focus on the case $q\ne 1$ only, since the other case is simpler.

Without loss of generality, we can assume that the limit in the left hand side of (59) is reached and it is finite, and hence there exists $C>0$ such that $E_n(\mu _{D_n}) \le C$ for every $n\in \mathbb {N}$. Then, by the second assertion of Lemma 4.2 there exists $C'>0$ such that $\#\partial D_n \le C'\sqrt{n}$ for every $n\in \mathbb {N}$, from which it follows that there exists a constant $C''>0$ such that

60 $$\begin{aligned} {\mathcal {H}}^1 (\partial H_n')<C'' \end{aligned}$$

for every $n\in \mathbb {N}$. Therefore, up to a non-relabeled subsequence, $\rho \chi _{H_n'}$ weakly converges in $BV_{\text {loc}}({\mathbb {R}}^2)$ to a function $g\in BV_{\text {loc}}({\mathbb {R}}^2)$. Since, up to extracting an extra non-relabeled subsequence, $\mu _{D_n}- \rho \chi _{H_n'} {\mathop {\rightharpoonup }\limits ^{*}} 0$ as proved in Lemma 5.1 and $\mu _{D_n} {\mathop {\rightharpoonup }\limits ^{*}} \rho \chi _D$ by hypothesis, then $g:=\rho \chi _D$ and $\rho \chi _{H_n'}{\mathop {\rightharpoonup }\limits ^{*}} \rho \chi _D$.

We observe that by (17), (33), and (55) we have that

61 $$\begin{aligned} E_n(\mu _{D_n}) = 2c_F {\mathcal {H}}^1 (\partial H_n')-c_S {\mathcal {H}}^1\left( \partial H_n' \cap \left\{ y_2=\frac{e_{FS} }{\sqrt{n}}-\frac{1}{2\sqrt{3n}}\right\} \cap O_n \right) , \end{aligned}$$

where $O_n$ is the oscillation set defined in (58). To check the validity of the above formula, one easily sees that the second term comes from those atoms that are connected with substrate atoms (for each such atom $a \in D_n$ the set $H_n'$ contains the segment $v(a) \cap \left\{ y_2=\frac{e_{FS} }{\sqrt{n}}-\frac{1}{2\sqrt{3n}}\right\}$ of length $1/\sqrt{n}$, see Fig. 4). For any other atom $a \in D_n$ one needs to see which neighboring atoms are missing. For example if all six neighbors are missing, then $\partial H_n'$ contains the triangle with sizes $1/\sqrt{n}$ contained in *v*(*a*), whose sides are parallel to vectors ${\varvec{t}}_1,{\varvec{t}}_2,{\varvec{t}}_2-{\varvec{t}}_1$, and on their unit normals the function $\Gamma$ takes the maximal value $2c_F$. If the atom $a \in D_n$ is missing the neighbor $a-{\varvec{t}}_2$, but neighbor $a+({\varvec{t}}_1-{\varvec{t}}_2)$ belongs to $D_n$, then $\partial H_n'$ contains the segment of size $1/\sqrt{n}$ in $v(a-{\varvec{t}}_2)$, parallel to the vector ${\varvec{t}}_1-{\varvec{t}}_2$. We associate one half of such segment to the atom *a*, while the other half to the atom $a+({\varvec{t}}_1-{\varvec{t}}_2)$, who is also missing a neighbor. The value of the function $\Gamma$ is also $2c_F$ on the unit normal to the vector ${\varvec{t}}_1-{\varvec{t}}_2$. On the other hand, if $a \in D_n$ is missing both of its neighbors $a-{\varvec{t}}_2$, $a+({\varvec{t}}_1-{\varvec{t}}_2)$, then $\partial H_n'$ certainly contains the segment of size $1/\sqrt{n}$ that belongs to *v*(*a*) and is parallel to the vector ${\varvec{t}}_1$. Therefore, the value of the function $\Gamma$ is $2c_F$ on the unit normal to the vector ${\varvec{t}}_1$.

Fix $\delta >0$ and consider in this proof the notation $y:=(y_1,y_2)\in \mathbb {R}^2$ for the coordinate of a point $y\in \mathbb {R}^2$. From (61), it easily follows that

$$\begin{aligned} E_n (\mu _{D_n})= & {} 2c_{F} {\mathcal {H}}^1 (\partial H_n' \cap \{y_2>\delta \})+2c_{F}{\mathcal {H}}^1 (\partial H_n' \cap \{0 \le y_2 \le \delta \})\\&-c_S {\mathcal {H}}^1\left( \partial H_n' \cap \left\{ y_2=\frac{e_{FS} }{\sqrt{n}}-\frac{1}{2\sqrt{3n}}\right\} \cap O_n \right) \\= & {} \int _{\partial H_n' \cap \{ y_2 >\delta \} }\Gamma (\nu _{H_n'}) d {\mathcal {H}}^1+2c_F{\mathcal {H}}^1 (\partial H_n' \cap \{0 \le y_2 \le \delta \})\\&-c_S {\mathcal {H}}^1\left( \partial H_n' \cap \left\{ y_2=\frac{e_{FS} }{\sqrt{n}}-\frac{1}{2\sqrt{3n}}\right\} \cap O_n \right) , \end{aligned}$$

where in the second equality we used the definition of $\Gamma$ (see (27)) to see that

$$\begin{aligned} \Gamma (\pm {\varvec{t}}_3)=\Gamma \left( \pm \frac{{\varvec{t}}_1+{\varvec{t}}_2}{\sqrt{3}}\right) =\Gamma \left( \pm \frac{{\varvec{t}}_2-2{\varvec{t}}_1}{\sqrt{3}}\right) =2 c_F. \end{aligned}$$

By Reshetnyak’s lower semicontinuity (Ambrosio et al. 2000, Theorem 2.38), we obtain that

$$\begin{aligned} \liminf _{n \rightarrow \infty } \int _{\partial H_n' \cap \{x_2>\delta \}} \Gamma (\nu _{H_n'}) d {\mathcal {H}}^1\ge & {} \liminf _{n \rightarrow \infty } \int _{\partial H_n'\cap B(R) \cap \{x_2>\delta \}} \Gamma (\nu _{H_n'}) d {\mathcal {H}}^1 \\\ge & {} \int _{\partial ^* D \cap B(R) \cap \{x_2>\delta \}} \Gamma (\nu _E) d{\mathcal {H}}^1, \end{aligned}$$

for every ball *B*(*R*) centered at the origin and with radius $R>0$, since $\chi _{H_n'}$ converges weakly* in $BV_{\text {loc}}({\mathbb {R}}^2)$ (and thus strongly in $L^1_{\text {loc}}$) to $\chi _D$, and hence, by letting $R \rightarrow \infty$,

62 $$\begin{aligned} \liminf _{n \rightarrow \infty } \int _{\partial H_n' \cap \{x_2>\delta \}} \Gamma (\nu _{H_n'}) d {\mathcal {H}}^1 \ge \int _{\partial ^* D \cap \{x_2>\delta \}} \Gamma (\nu _D ) d{\mathcal {H}}^1. \end{aligned}$$

We claim that for all $\delta >0$ small enough

63 $$\begin{aligned}&\liminf _{n \rightarrow \infty } \left[ 2c_F {\mathcal {H}}^1 (\partial H_n' \cap \{0 \le y_2 \le \delta \})-c_S {\mathcal {H}}^1\left( \partial H_n' \cap \left\{ y_2=\frac{e_{FS} }{\sqrt{n}}-\frac{1}{2\sqrt{3n}}\right\} \cap O_n \right) \right] \nonumber \\&\quad \ge \left( 2c_F-{ \frac{c_S}{q}} \right) {\mathcal {H}}^1(\partial ^*D \cap \{y_2=0\}) \end{aligned}$$

and we notice that from (62) and (63) we obtain

$$\begin{aligned} \liminf _{n \rightarrow \infty } E_n(\mu _{D_n})\ge & {} \int _{\partial ^*D \cap \{x_2>\delta \}} \Gamma (\nu _D) d{\mathcal {H}}^1+\left( 2c_F-{\frac{c_S}{q}}\right) {\mathcal {H}}^1(\partial ^*D \cap \{x_2=0\}), \end{aligned}$$

from which (59) directly follows by letting $\delta \rightarrow 0$. To prove the claim (63), we fix $\delta >0$, we introduce the Borel measures $\kappa _{1,n}, \kappa _{2,n}$, and $\kappa _n$ defined by

$$\begin{aligned} \kappa _{1,n} (B):= & {} {\mathcal {H}}^1 (\partial H_n' \cap \{0 \le y_2 \le \delta \}\cap B),\\ \kappa _{2,n}(B):= & {} {\mathcal {H}}^1\left( \partial H_n' \cap \left\{ y_2=\frac{e_{FS} }{\sqrt{n}}-\frac{1}{2\sqrt{3n}}\right\} \cap O_n \cap B\right) ,\\ \kappa _n (B):= & {} 2c_F\kappa _{1,n}(B)-c_S{\kappa _{2,n}}(B), \end{aligned}$$

for every $B \in {\mathcal {B}} ({\mathbb {R}}^2)$, where ${\mathcal {B}} (A)$ for a set *A* denotes the Borel $\sigma$-algebra on *A*, and we consider the sets

$$\begin{aligned} Q_{M}:&=[-M,M] \times [0,\delta ], \quad \mathring{Q}_M:=(-M,M) \times [0,\delta ],\quad \text {and}\quad \\&Q_M^c:=(\mathbb {R}\times [0,\delta ])\backslash Q_M. \end{aligned}$$

We divide the proof in three steps:

**Step 1.** In this step we prove that for every $M>0$ we have that

64 $$\begin{aligned} \liminf _{n \rightarrow \infty } \kappa _n(Q_M) \ge (2c_F-\frac{c_S}{q}){\mathcal {H}}^1(\partial ^*D \cap \{x_2=0\}\cap Q_M). \end{aligned}$$

By (60) we conclude that, up to extracting a non-relabeled sub-subsequence (for an arbitrary a priori chosen subsequence in *n*), for fixed $M>0$, there exist Borel measures $\kappa _1^M$ and $\kappa _2^M$ such that $\kappa _{1,n}|_{Q_M} {\mathop {\rightharpoonup }\limits ^{*}} \kappa _1^M$ and $\kappa _{2,n}|_{Q_M} {\mathop {\rightharpoonup }\limits ^{*}} \kappa _2^M$. Consequently, $\kappa _n|_{Q_M} {\mathop {\rightharpoonup }\limits ^{*}} 2c_F \kappa _1^M-c_S \kappa _2^M$. By $\mu _n^M$, we denote the measure

$$\begin{aligned} \mu _n^M(\cdot )={\mathcal {H}}^1 (\{ y_2 = e_{FS}/{\sqrt{n}}-1/(2\sqrt{3n})\}\cap Q_M \cap \cdot ). \end{aligned}$$

By using Lemma 5.2 (applied to measures $\kappa _{2,n}^M$ and $\mu _n^M$), we conclude that $\kappa _2^M$ is absolutely continuous with respect to the Borel measure

65 $$\begin{aligned} \mu ^M(\cdot )={\mathcal {H}}^1 (\{ y_2 =0\}\cap Q_M \cap \cdot ) \end{aligned}$$

and we denote its density with respect to $\mu ^M$ by $\zeta _2^M$. The measure $\kappa _1^M$ might not be absolutely continuous with respect to $\mu _M$ . We denote the density of its absolutely continuous part with respect to $\mu ^M$ by $\zeta _1^M$. To conclude the proof of (64) we need to show

66 $$\begin{aligned} 2c_F\zeta _1^M( y_1)-c_S\zeta _2^M( y_1)\ge & {} 2c_F-\frac{c_S}{q}, \text { for } (y_1,0) \in \partial ^{*} D \cap \mathring{Q}_M, \end{aligned}$$

67 $$\begin{aligned} 2c_F\zeta _1^M( y_1)-c_S\zeta _2^M( y_1)\ge & {} 0, \text { for } {\mathcal {H}}^1\text { a.e. } ( y_1,0) \in (\partial S{\setminus }\partial ^{*} D )\cap \mathring{Q}_M. \end{aligned}$$

We begin by showing (66). Take $y'=(y'_1,0) \in \partial ^* D \cap \mathring{Q}_M$ and denote by $Q_{\varepsilon }(y')$ the square centered at $y'$ with edges of size $\varepsilon$ parallel to the coordinate axes and $Q^{+}_{\varepsilon }(y'):=Q_{\varepsilon }( y')\cap \{y_2>0\}$. We assume $\varepsilon >0$ is small enough such that $Q^{+}_{\varepsilon }(y') \subset \mathring{Q}_M$. By standard properties (see, e.g., Ambrosio et al. 2000, Example 3.68) we conclude that

$$\begin{aligned} \lim _{\varepsilon \rightarrow 0} \frac{1}{\varepsilon ^2} \int _{Q^{+}_{\varepsilon }(y') } |\chi _{D }(z)-1|dz=0. \end{aligned}$$

Since $\chi _{H_n'} \rightarrow \chi _{D }$ as $n\rightarrow \infty$ in $L^1(Q^{+}_{\varepsilon }(y'))$ for $\varepsilon >0$ fixed we conclude that

$$\begin{aligned} \lim _{\varepsilon \rightarrow 0} \lim _{n \rightarrow \infty } \frac{1}{\varepsilon ^2} \int _{Q^{+}_{\varepsilon }(y') } |\chi _{H_n'}(z)-1|dz=\lim _{\varepsilon \rightarrow 0}\frac{1}{\varepsilon ^2} \int _{Q^{+}_{\varepsilon }(y') } |\chi _{D }(z)-1|dz=0. \end{aligned}$$

Thus, for every $0<\alpha <1$ there exists $0<\varepsilon _0< \frac{\delta }{2}$ such that

$$\begin{aligned} \liminf _{n \rightarrow \infty } |H_n' \cap Q^{+}_{\varepsilon }(y' )|\ge \frac{\alpha }{2}\varepsilon ^2, \quad \forall \varepsilon < \varepsilon _0. \end{aligned}$$

Next we define the sets $Q^0_{\varepsilon }(y'):= Q_{\varepsilon }(y') \cap \{y_2=0\}$. We have that

68 $$\begin{aligned} {\liminf _{n \rightarrow \infty } {\mathcal {H}}^1\left( \{(y_1,0) \in Q^0_{\varepsilon }(y'): (\{y_1\} \times {\mathbb {R}}^{+} )\cap H_n' \ne \emptyset \} \right) \ge \varepsilon \alpha . } \end{aligned}$$

We look at the “lower polygonal curve” $c_{\ell , n}$ of $H'_n$ defined by:

$$\begin{aligned} c_{\ell , n}:=\bigcup _{x \in H_n'} p_{\ell ,n}(x), \end{aligned}$$

where $p_{\ell ,n}:=(p_{\ell ,n}^1(x),p_{\ell ,n}^2(x)) \in \partial H_n'$ is the projection function given by

$$\begin{aligned} p_{\ell ,n}^1(x):=x_1, \quad p_{\ell ,n}^2(x):=\inf \{y_2>0:(x_1,y_2) \in H_n'\} \end{aligned}$$

for every $x=(x_1,x_2) \in H_n'$. Then, $\{(y_1,0) \in Q^0_{\varepsilon }(y'): (\{y_1\} \times {\mathbb {R}}^{+} )\cap H_n' \ne \emptyset \}= \pi _1( c_{\ell ,n})\cap Q^0_{\varepsilon }(y')$, where $\pi _1$ is the projection of points of $\mathbb {R}^2$ onto $\{y_2=0\}$ and hence, by (68)

69 $$\begin{aligned} \liminf _{n \rightarrow \infty } {\mathcal {H}}^1\left( \pi _1( c_{\ell ,n})\cap Q^0_{\varepsilon }(y')\right) \ge \varepsilon \alpha . \end{aligned}$$

Furthermore, notice that $c_{\ell ,n}$ is a union of segments of length $1/\sqrt{n}$, each of them associated with an atom belonging to $D_n$. In the cube $Q_{\varepsilon }(y')$ there are $k_1(n)$, $k_1(n) \le \lceil \frac{\sqrt{n}\varepsilon }{q} \rceil$, horizontal segments of size $1/\sqrt{n}$ that both belong to $c_{\ell ,n}$ and correspond to an atom of $D_n$ bonded with a substrate atom. Thus, they contribute by $\frac{k_1(n)}{\sqrt{n}}\le \lceil \frac{\sqrt{n}\varepsilon }{q} \rceil \frac{1}{\sqrt{n}}$ to the length of $\pi _1( c_{\ell ,n})\cap Q^0_{\varepsilon }(y')$. We have that $\lim _{n \rightarrow \infty } \lceil \frac{\sqrt{n}\varepsilon }{q} \rceil \frac{1}{\sqrt{n}}=\frac{\varepsilon }{q}$. We denote by $k_2(n)$ the number of segments in $c_{\ell ,n}$ of size $1/\sqrt{n}$ that are not computed in the $k_1(n)$ segments. From (69) it follows that

70 $$\begin{aligned} \liminf _{n \rightarrow \infty }\kappa _n(Q_{\varepsilon } (y'))\ge & {} \liminf _{n \rightarrow \infty } \left( \frac{k_1(n)}{\sqrt{n}}(2c_F-c_S)+\frac{k_2(n)}{\sqrt{n}} 2c_F\right) \nonumber \\\ge & {} { \left( 1/q (2c_F-c_S)+(\alpha -1/q)2c_F \right) \varepsilon .} \end{aligned}$$

Next we take a sequence in $(\varepsilon )$, still denoted by $(\varepsilon )$ such that for each member of the sequence we have $\kappa _1(\partial Q_{\varepsilon }(y'))=\kappa _2(\partial Q_{\varepsilon }(y'))=0$ . By the standard properties of measures (see Evans and Gariepy 2015, Section 1.6.1, Theorem 1) and (70) we have

$$\begin{aligned} 2c_F \zeta _1^M(y')-c_S \zeta _2^M(y')= & {} \lim _{\varepsilon \rightarrow 0} \frac{2c_F \kappa _1^M(Q_{\varepsilon }(y'))-c_S \kappa _2^M(Q_{\varepsilon }(y'))}{\varepsilon } \\= & {} \lim _{\varepsilon \rightarrow 0} \lim _{n \rightarrow \infty } \frac{\kappa _n(Q_{\varepsilon } (y'))}{\varepsilon } \\\ge & {} { 1/q (2c_F-c_S)+(\alpha -1/q)2c_F}. \end{aligned}$$

By letting $\alpha \rightarrow 1$ we have (66).

It remains to show (67). Let $y'=(y_1',0) \in \mathring{Q}_M \backslash \partial ^{*} D$. Notice that by standard properties of BV functions ${\mathcal {H}}^1$ a.e. $(y_1,0)$ that does not belong to $\partial ^{*} E$, belongs to the set of density zero for *D* (see Ambrosio et al. 2000, Theorem 3.61), i.e.,

$$\begin{aligned} \lim _{\varepsilon \rightarrow 0} \lim _{n \rightarrow \infty } \frac{1}{\varepsilon ^2} \int _{Q^{+}_{\varepsilon }(y') } \chi _{H_n'}(y)dy=\lim _{\varepsilon \rightarrow 0}\frac{1}{\varepsilon ^2} \int _{Q^{+}_{\varepsilon }(y') }\chi _{D }(y)dy=0. \end{aligned}$$

Thus, for each $\alpha >0$ there exits $\varepsilon _0>0$ such that

71 $$\begin{aligned} \limsup _{n \rightarrow \infty } |H_n' \cap Q^{+}_{\varepsilon }(y')|\le \alpha \varepsilon ^2, \quad \forall \varepsilon < \varepsilon _0. \end{aligned}$$

We need to pay attention to the atoms $y'$ that are bonded with substrate atoms, whose deficiency contribution (recall 19) can be negative and as low as $2c_F-c_S$.

The proof consists in showing that for *n* large enough the total “energy deficiency” on the cube $Q_{\varepsilon } (y')$ is actually positive, since there is “not much of set *D*” in the cube $Q_{\varepsilon } (y')$. We define

$$\begin{aligned} K_{n,\varepsilon }(y'):= \frac{\partial {\mathcal {L}}_{FS}}{\sqrt{n}}\cap Q_{\varepsilon } (y') \cap \frac{D_n}{\sqrt{n}} \end{aligned}$$

Fix $a_0\in K_{n,\varepsilon }(y')$ and denote by $a_{-1}$ and $a_{1}$ the closest points to $a_0$ in $\partial {\mathcal {L}}_{F}/\sqrt{n}$ on the left and on the right of $a_0$, respectively. We consider the set

$$\begin{aligned} {{\widetilde{O}}}_n^{a_0}:=\bigcup _{i=-1,0,1}{\widetilde{O}}_{n}^{a_0,i}, \end{aligned}$$

where ${{\widetilde{O}}}_{n}^{a_0,-1}:=O_n^{a_{-1},+}$, $\widetilde{O}_{n}^{a_0,1}:=O_n^{a_{1},-}$, and $\widetilde{O}_{n}^{a_0,0}:=O_n^{a_{0}}$, and we denote its projection onto $\partial S$ by $P^{a_0}_n$. Notice that ${\mathcal {H}}^1(P^{a_0}_n)=2/\sqrt{n}$. We claim that

$$\begin{aligned} \limsup _{n \rightarrow \infty } {\mathcal {H}}^1 \left( \bigcup _{a_0 \in \tilde{K}_{n, \varepsilon }(y')} P^{a_0}_{n}\right) \le 16\alpha \varepsilon \end{aligned}$$

where

$$\begin{aligned} {{\widetilde{K}}}_{n,\varepsilon }(y'):= \left\{ a_0\in K_{n,\varepsilon }(y')\,:\, \exists i\in \{-1,0,1\}\text { such that}\, |{{\widetilde{O}}}_{n}^{a_0,i}\cap H_n'\cap Q_{\varepsilon } (y')|>\frac{\varepsilon }{8\sqrt{n}} \right\} . \end{aligned}$$

Indeed, as a consequence of (71) we have

72 $$\begin{aligned} \#{{\widetilde{K}}}_{n,\varepsilon }(y') \le 8\alpha \varepsilon \sqrt{n}+1 \end{aligned}$$

and hence, by (72) we have

73 $$\begin{aligned} \sum _{a_0 \in {\widetilde{K}}_{n,\varepsilon }(y')}\kappa _{n}^M ({{\widetilde{O}}}_n^{a_0}) \ge -|2c_F-c_S|(8\alpha \varepsilon \sqrt{n}+1) \frac{1}{\sqrt{n}}. \end{aligned}$$

We now fix $a_0 \in K_{n, \varepsilon }(y') \backslash \widetilde{K}_{n, \varepsilon }(y')$ such that $a_0$ is neither the first left nor the last right atom in $K_{n,\varepsilon }(y')$ and show by a simple analysis of the atoms $a_i$, $i=-1,0,1$ that

74 $$\begin{aligned} {\mathcal {H}}^1(\partial H_n'\cap Q_{\varepsilon } (y') \cap \widetilde{O}_n^{a_0}) \ge \frac{3}{\sqrt{n}} \end{aligned}$$

which immediately implies that for $a_0 \in K_{n, \varepsilon }(y') \backslash {{\widetilde{K}}}_{n, \varepsilon }(y')$ the energy contribution of the strip $Q_{\varepsilon } (y') \cap \widetilde{O}_n^{a_0}$ for every $\varepsilon >0$ is positive and so,

75 $$\begin{aligned} \sum _{a_0 \in K_{n,\varepsilon }(y')\backslash {\widetilde{K}}_{n,\varepsilon }(y')}\kappa _{n}^M ({{\widetilde{O}}}_n^{a_0}) \ge (6c_F-c_S) \frac{\# (K_{n,\varepsilon }(y')\backslash {\widetilde{K}}_{n,\varepsilon }(y') )}{\sqrt{n}}\ge 0. \end{aligned}$$

To prove (74), we analyze the three possible cases:

1. both of the strips ${{\widetilde{O}}}_{n}^{a_0,-1}$ and ${{\widetilde{O}}}_{n}^{a_0,+1}$ have empty intersection with $H_n'$;
2. one of the strips ${{\widetilde{O}}}_{n}^{a_0,-1}$ and ${{\widetilde{O}}}_{n}^{a_0+1}$ has empty intersection with $H_n'$;
3. none of the strips ${{\widetilde{O}}}_{n}^{a_0,-1}$ and ${{\widetilde{O}}}_{n}^{a_0,+1}$ has empty intersection with $H_n'$;

In the first case we have that $a_0$ does not have neighbors and hence, there is a part of $\partial H_n'$ of length $3/\sqrt{n}$ (perimeter of the equilateral triangle with side of size $1/\sqrt{n}$) that surrounds $a_0$, i.e., belongs to $v(a_0)\cap \partial H_n'$. This proves (74) in the case of (1). For the second case we suppose without loss of generality that the interior of the strip of ${{\widetilde{O}}}_{n}^{a_0,-1}$ has empty intersection with $H_n'$. We take the atom $x_1^r$ that belongs to $D_n\cap \sqrt{n} \overline{{{\widetilde{O}}}_{n}^{a_0,+1}}$that is the lowest and neighbor of $a_0$ and the atom $x_2^r \in D_n\cap \sqrt{n} \overline{{{\widetilde{O}}}_{n}^{a_0,+1}}$ that does not have at least one of the two of his upper neighbors. If the first atom does not exist it is easy to see that (74) is satisfied, and the second one exists by the fact that $a_0 \in K_{n, \varepsilon }(y') \backslash {{\tilde{K}}}_{n, \varepsilon }(y')$. It is easy to see that (74) is satisfied also in this case since we have contribution of $2/\sqrt{n}$ from $v(a_0) \cap \partial H_n'$, where *v* is defined in (53), and at least $1/(2\sqrt{n})$ from

$$\begin{aligned} \left( v(x_1^r) \cup v\left( x_1^r-{\varvec{t}}_2\right) \cup v\left( x_1^r+({\varvec{t}}_1-{\varvec{t}}_2)\right) \right) \cap \widetilde{O}_{n}^{a_0,+1} \cap \partial H_n', \end{aligned}$$

and at least $1/(2\sqrt{n})$ from $v(x_2^r)\cap \widetilde{O}_{n}^{a_0,+1} \cap \partial H_n'$; in the case when $x_1^r=x_2^r$ we have the contribution of at least $1/\sqrt{n}$ from

$$\begin{aligned} \left( v(x_1^r) \cup v\left( x_1^r-{\varvec{t}}_2\right) \cup v\left( x_1^r+({\varvec{t}}_1-{\varvec{t}}_2)\right) \right) \cap \widetilde{O}_{n}^{a_0,-1} \cap \partial H_n'. \end{aligned}$$

In a similar way in the third case, we find atoms $x_1^l,x_2^l \in D_n \cap \sqrt{n} \overline{{{\widetilde{O}}}_{n}^{a_0,+1}}$ and $x_1^r,x_2^r \in D_n \cap \sqrt{n} \overline{\widetilde{O}_{n}^{a_0,+1}}$ for which there exist contribution of $1/\sqrt{n}$ coming from $v(a_0) \cap \partial H_n'$, $1/\sqrt{n}$ coming from

$$\begin{aligned}\Bigg [\left( v(x_1^r) \cup v\left( x_1^r-{\varvec{t}}_2\right) \cup v\left( x_1^r+({\varvec{t}}_1-{\varvec{t}}_2)\right) \right) \cap&\widetilde{O}_{n}^{a_0,+1}\cap \partial H_n'\Bigg ]\\&\cup (v(x_2^r)\cap {{\widetilde{O}}}_{n}^{a_0,+1} \cap \partial H_n'), \end{aligned}$$

and $1/\sqrt{n}$ coming from

$$\begin{aligned}\Bigg [ \left( v(x_1^l) \cup v\left( x_1^l-{\varvec{t}}_2\right) \cup v\left( x_1^l+({\varvec{t}}_1-{\varvec{t}}_2)\right) \right)&\cap \widetilde{O}_{n}^{a_0,-1} \cap \partial H_n' \Bigg ]\\&\cup (v(x_2^l)\cap {{\widetilde{O}}}_{n}^{a_0,-1} \cap \partial H_n'). \end{aligned}$$

The rest of the energy deficiency that is inside the strip is positive. From (73) and (75), we conclude that

$$\begin{aligned} 2c_F \zeta ^M_1(y')-c_S \zeta ^M_2(y')= & {} \lim _{\varepsilon \rightarrow 0} \frac{2c_F \kappa _1^M(Q_{\varepsilon }(y'))-c_S \kappa _2^M(Q_{\varepsilon }(y'))}{\varepsilon } \\= & {} \lim _{\varepsilon \rightarrow 0} \lim _{n \rightarrow \infty } \frac{\kappa _n(Q_{\varepsilon } (y'))}{\varepsilon } \\\ge & {} -8|2c_F-c_S| \alpha . \end{aligned}$$

By letting $\alpha \rightarrow 0$, (67) follows.

**Step 2.** In this step, we deduce (63) from the inequalities (66) and (67) proved in Step 1. It suffices to show that for every $\varepsilon >0$ there exist $M_0>0$ and $n_0 \in {\mathbb {N}}$ such that

76 $$\begin{aligned} \kappa _n (Q_M^c) \ge -\varepsilon \end{aligned}$$

for every $M \ge M_0$, $n \ge n_0$.To establish (76) fix $\varepsilon >0$ and choose $M_0>0$ and

$$\begin{aligned} n_0 > \frac{16}{\varepsilon ^2c_S^2}+1 \end{aligned}$$

large enough so that the following three assertions hold:

1. $$|D \cap Q_{M_0}^c| \le \frac{1}{48c_S}\varepsilon \delta $$,
2. $$\left| (D \cap Q_{M_0}) \Delta (H_n\cap Q_{M_0})\right| \le \frac{1}{48c_S} \varepsilon \delta $$, $$\forall n \ge n_0$$,
3. $$|H_n \Delta H_n'| \le \frac{1}{48c_S}\varepsilon \delta $$, $$\forall n \ge n_0$$.

Notice that such $M_0$ and $n_0$ exist since (1) is trivial for large $M_0$, (2) follows from the $BV_{\text {loc}}$-convergence of $\rho \chi _{H_n}$ to $\rho \chi _D$ , and (3) is a consequence of (56). By (2) and (3) and the fact that

$$\begin{aligned} |H_n|=|D |=\frac{\sqrt{3}}{2}, \end{aligned}$$

we have that

77 $$\begin{aligned} |H_n' \cap Q_M^c| \le \frac{1}{16c_S} \varepsilon \delta , \quad \forall M \ge M_0, \forall n \ge n_0. \end{aligned}$$

We define

$$\begin{aligned} K_{n,M}:= \frac{\partial {\mathcal {L}}_{FS}}{\sqrt{n}}\cap Q_M^c \cap \frac{D_n}{\sqrt{n}} . \end{aligned}$$

Following the same idea of the previous step the proof consists in using the fact that “there is not much of the set *D* outside $Q_M$” and hence, the energy deficiency outside $Q_M$ is small, for *n* large enough.

From (77), it follows that the set ${{\widetilde{K}}}_{n,M}$ defined by

$$\begin{aligned} {{\widetilde{K}}}_{n,M}:= \left\{ a\in K_{n,M}\,:\, \exists \alpha \in \{-1,+1\}\text { such that}\, |\widetilde{O}_{n}^{a,\alpha }\cap H_n'\cap ({\mathbb {R}} \times [0,\delta ])|>\frac{\delta }{8\sqrt{n}} \right\} \\ \end{aligned}$$

is such that

$$\begin{aligned} \#{{\widetilde{K}}}_{n,M} \le \frac{1}{2c_S} \sqrt{n} \varepsilon +2, \end{aligned}$$

and hence

78 $$\begin{aligned} {\mathcal {H}}^1\left( \bigcup _{a \in {{\widetilde{K}}}_{n,M}} \left( a-\frac{1}{2 \sqrt{n}}, a+\frac{1}{2 \sqrt{n}}\right) \right) \le \frac{\varepsilon }{c_S} \end{aligned}$$

for every $n \ge n_0$. Since following the same argumentation of the previous step the energy deficiency associated with points in $K_{n,M} \backslash {{\widetilde{K}}}_{n,M}$ is shown to be positive, from (78) we easily conclude (76) for every $M \ge M_0$ and $n \ge n_0$.

**Step 3**. Claim (63) is an easy consequence of Step 1 and Step 2. More precisely, from Step 1 and Step 2 we have that for every $\varepsilon >0$ there exists $M_0>0$ such that

$$\begin{aligned} \liminf _{n\rightarrow \infty } \kappa _n (\mathbb {R}^2)\ge & {} \liminf _{n \rightarrow \infty } \kappa _n(Q_M) +\liminf _{n \rightarrow \infty } \kappa _n(Q_M^c)\\\ge & {} \left( 2c_F-\frac{c_S}{q}\right) {\mathcal {H}}^1(\partial ^*E\cap \{x_2=0\}\cap Q_M)-\varepsilon \end{aligned}$$

for any $M \ge M_0$, where we used (64) and (76). By letting $M \rightarrow \infty$ and using arbitrariness of $\varepsilon >0$ we obtain (63).

## Upper Bound

The proof of the upper bound follows from the arguments of Au Yeung et al. (2012) by paying extra care to the contact with the substrate.

### Theorem 6.1

For every set $D \subset \mathbb {R}^2{\setminus }S$ of finite perimeter such that $|D|=1/\rho$, there exists a sequence of configurations $D_n \in {\mathcal {C}}_n$ such that the corresponding associated empirical measures $\mu _{D_n}$ weakly* converge to $\rho \chi _D$ and $I_n (\mu _{D_n}) \rightarrow {\mathcal {E}}(D)$.

### Proof

The proof is divided in 5 steps.

**Step 1 (Approximation by bounded smooth sets).** In this step we claim that: If $E \subset \mathbb {R}^2{\setminus }S$ is a set of finite perimeter with $|E|=1/\rho$, then there exists a sequence of sets $(E_j)_{j \in {\mathbf {N}}}$ with $E_j \subset \mathbb {R}^2{\setminus }S$ for $j \in {\mathbf {N}}$ such that the following assertions hold:

(i) $$|E_j|= 1/\rho $$;
(ii) $E_j$ are bounded;
(iii) there exist sets $E_j' \subset {\mathbb {R}}^2$ of class ${\mathcal {C}}^{\infty }$ such that $E_j=E_j' \cap (\mathbb {R}^2{\setminus }{\bar{S}})$;
(iv) $$|E_j \Delta E| \rightarrow 0$$ as $$j \rightarrow \infty $$;
(v) $$|D \chi _{E_j}| (\mathbb {R}^2{\setminus }{\bar{S}}) \rightarrow | D \chi _E | (\mathbb {R}^2{\setminus }{\overline{S}})$$ as $$j \rightarrow \infty $$;
(vi) 79 $$\begin{aligned} \int _{\partial ^* E_j \cap (\mathbb {R}^2{\setminus }{\bar{S}})} \Gamma (\nu _{E_j} ) d {\mathcal {H}}^1 \rightarrow \int _{\partial ^* E \cap (\mathbb {R}^2{\setminus }{\overline{S}})} \Gamma (\nu _{E} ) d {\mathcal {H}}^1 \text { as } j \rightarrow \infty ; \end{aligned}$$
(vii) 80 $$\begin{aligned} {\mathcal {H}}^1 (\partial ^{*} E_j \cap \partial S) \rightarrow {\mathcal {H}}^1 (\partial ^{*} E \cap \partial S) \text { as } j \rightarrow \infty . \end{aligned}$$

We now construct the sequence of sets $(E_j)_{j \in {\mathbf {N}}}$ that satisfy (ii)–(vii) and observe that then (i) is easily obtained by scaling. Let $E' \subset {\mathbb {R}}^2$ be the set determined from *E* by reflection over $\partial S$ and note that

81 $$\begin{aligned} {\mathcal {H}}^1(\partial ^*E'\cap \partial S)=0. \end{aligned}$$

By Maggi (2012, Theorem 13.8 and Remark 13.9) we find smooth bounded open sets $E_j'\subset {\mathbb {R}}^2$ that satisfy $| E_j' \Delta E'|\rightarrow 0$ and

82 $$\begin{aligned} |D\chi _{ E_j'}| ({\mathbb {R}}^2) \rightarrow |D\chi _{E'}| ({\mathbb {R}}^2). \end{aligned}$$

We define $E_j:= E_j' \cap (\mathbb {R}^2{\setminus }{\bar{S}})$ and we claim that the sets $E_j$ satisfy (ii)-(vii).

We begin by noticing that (ii)-(iv) are trivial. To prove assertion (v) we begin to observe that

83 $$\begin{aligned} |D \chi _{E'}|(\mathbb {R}^2{\setminus }{\overline{S}})\le & {} \liminf _{j \rightarrow \infty } |D \chi _{E_j'}|(\mathbb {R}^2{\setminus }{\overline{S}}), \end{aligned}$$

84 $$\begin{aligned} |D \chi _{E'}|(S)\le & {} \liminf _{j \rightarrow \infty } |D \chi _{E_j'}|(S), \end{aligned}$$

85 $$\begin{aligned} |D \chi _{E'}|(\partial S)=0\le & {} \liminf _{j \rightarrow \infty } |D \chi _{E_j'}|(\partial S), \end{aligned}$$

where we used (81) and (82), and hence,

$$\begin{aligned}&|D \chi _{E'}|(\mathbb {R}^2)=|D \chi _{E'}|(\mathbb {R}^2{\setminus }{\overline{S}})+|D \chi _{E'}|(S)+ |D \chi _{E'}|(\partial S)\\&\quad \le \liminf _{j \rightarrow \infty } |D \chi _{E_j'}|(\mathbb {R}^2{\setminus }{\overline{S}})+ \liminf _{j \rightarrow \infty } |D \chi _{E_j'}|(S)+ \liminf _{j \rightarrow \infty } |D \chi _{E_j'}|(\partial S)\\&\quad \le \liminf _{j \rightarrow \infty } |D \chi _{E_j'}|(\mathbb {R}^2)=|D \chi _{E'}|(\mathbb {R}^2). \end{aligned}$$

Since this can be done on an arbitrary subsequence we have

86 $$\begin{aligned} |D \chi _{E}|(\mathbb {R}^2{\setminus }{\overline{S}})= |D \chi _{E'}|(\mathbb {R}^2{\setminus }{\overline{S}})= \lim _{j \rightarrow \infty } |D \chi _{E_j'}|(\mathbb {R}^2{\setminus }{\overline{S}})= \lim _{j \rightarrow \infty } |D \chi _{E_j}|(\mathbb {R}^2{\setminus }{\overline{S}}), \end{aligned}$$

since by the definition of $E'$ and $E_j$ we have that $D \chi _{E'}(A) = D \chi _{E}(A)$ and $D\chi _{E_j'}(A) = D \chi _{E_j}(A)$ for every $A\in {\mathcal {B}}(\mathbb {R}^2{\setminus }{\overline{S}})$.

Assertion (vi) is a direct consequence of the Reshetnyak continuity theorem (Ambrosio et al. 2000, Theorem 2.39).

To prove assertion (vii) we will first claim that for almost every $M>0$

87 $$\begin{aligned} {\mathcal {H}}^1 (\partial E_j \cap (-M,M) \times \{0\}) \rightarrow {\mathcal {H}}^1 (\partial ^{*} E\cap (-M,M)\times \{0\}). \end{aligned}$$

To prove claim (87) we observe that for almost every $M \ge 0$ we have

$$\begin{aligned} |D \chi _{E'}| \left( (\{-M\}\times {\mathbb {R}} )\cup (\{M\} \times {\mathbb {R}})\right) =0. \end{aligned}$$

In fact the set of $M\ge 0$ where this condition is not satisfied is at most countable. As in the proof of (v), we conclude that for all such $M \ge 0$ we have

88 $$\begin{aligned} |D\chi _{ E_j}|\left( (-\infty , -M) \times {\mathbb {R}}^+\right)\rightarrow & {} |D\chi _{E}|\left( (-\infty , -M) \times {\mathbb {R}}^+\right) , \end{aligned}$$

89 $$\begin{aligned} |D\chi _{E_j}|\left( (M, +\infty ) \times {\mathbb {R}}^+\right)\rightarrow & {} |D\chi _{E}|\left( ( M, +\infty ) \times {\mathbb {R}}^+\right) , \end{aligned}$$

90 $$\begin{aligned} |D\chi _{E_j}|\left( ( -M, M ) \times {\mathbb {R}}^+\right)\rightarrow & {} |D\chi _{E}|\left( (-M, M ) \times {\mathbb {R}}^+\right) . \end{aligned}$$

We then notice that (87) is a consequence of continuity of traces and (90).

We now make the second claim that

91 $$\begin{aligned}&\lim _{M \rightarrow \infty } \lim _{j \rightarrow \infty } {\mathcal {H}}^1\left( \partial E_j \cap (-\infty , -M) \times \{0\} \right) = 0, \end{aligned}$$

92 $$\begin{aligned}&\lim _{M \rightarrow \infty } \lim _{j \rightarrow \infty } {\mathcal {H}}^1\left( \partial E_j \cap ( M, +\infty ) \times \{0\} \right) = 0, \end{aligned}$$

where the limit in *M* is taken over sequence of *M* that satisfy (88)–(90), which together with (87) yields (vii) since

$$\begin{aligned} {\mathcal {H}}^1 (\partial ^{*} E \cap \partial S)= & {} \lim _{M \rightarrow \infty } {\mathcal {H}}^1 (\partial ^{*} E\cap (-M,M)\times \{0\})\\= & {} \lim _{M \rightarrow \infty }\lim _{j \rightarrow \infty } {\mathcal {H}}^1 (\partial E_j \cap (-M,M) \times \{0\}) \\= & {} \lim _{j \rightarrow \infty } {\mathcal {H}}^1 (\partial E_j \cap \partial S). \end{aligned}$$

We prove only (91), since (92) goes in an analogous way. It is enough to show that

93 $$\begin{aligned} {\mathcal {H}}^1 \left( \partial E_j \cap (-\infty ,-M) \times \{0\}\right) \le |D \chi _{ E_j}| ((-\infty ,-M)\times {\mathbb {R}}^+), \end{aligned}$$

and apply (88). Estimate (93) can be seen by taking $\varphi \in C_c({\mathbb {R}}^2)$, $\varphi ={\varvec{t}}_3$ on some open set *F* such that $E_j \cap (-\infty , -M) \times {\mathbb {R}}^+ \subset \subset F$, in the identity

$$\begin{aligned} \int _{\partial \left( E_j \cap (-\infty , -M) \times {\mathbb {R}}^+\right) } \langle \nu _{E_j \cap (-\infty , -M) \times {\mathbb {R}}^+},\varphi \rangle dx= & {} \int _{F} \langle D\chi _{E_j \cap \left( (-\infty , -M) \times {\mathbb {R}}^+\right) },\varphi \rangle dx \\= & {} \int _F \chi _{E_j \cap \left( (-\infty , -M) \times {\mathbb {R}}^+\right) } \text {div} \varphi dx =0. \end{aligned}$$

From this it follows that

94 $$\begin{aligned} \int _{\partial \left( E_j \cap (-\infty , -M) \times {\mathbb {R}}^+\right) \cap \{x_2>0\}} \langle&\nu _{E_j \cap (-\infty , -M) \times {\mathbb {R}}^+}, {\varvec{t}}_3\rangle d {\mathcal {H}}^1\nonumber \\&={\mathcal {H}}^1 \left( \partial \left( E_j \cap (-\infty , -M) \times {\mathbb {R}}^+\right) \cap \{x_2=0\}\right) , \end{aligned}$$

where we used the fact that

$$\begin{aligned} \nu _{E_j \cap (-\infty , -M) \times {\mathbb {R}}^+}=-{\varvec{t}}_3,\quad {\mathcal {H}}^1 \text { almost everywhere on } x_2=0. \end{aligned}$$

Therefore, by (94) and since

$$\begin{aligned} \nu _{E_j \cap (-\infty , -M) \times {\mathbb {R}}^+}={\varvec{t}}_1,\quad {\mathcal {H}}^1 \text { almost everywhere on } x_1=-M. \end{aligned}$$

we obtain

95 $$\begin{aligned}&{\mathcal {H}}^1 \left( \partial \left( E_j \cap (-\infty , -M) \times {\mathbb {R}}^+\right) \cap \{x_2=0\} \right) \nonumber \\&\le {\mathcal {H}}^1 \left( \partial \left( E_j \cap (-\infty , -M) \times {\mathbb {R}}^+\right) \cap (-\infty ,-M)\times {\mathbb {R}}^+ \right) . \end{aligned}$$

Notice that

96 $$\begin{aligned} \partial \left( E_j \cap ((-\infty , -M) \times {\mathbb {R}}^+)\right) \cap (-\infty ,-M)\times {\mathbb {R}}^+ =\partial E_j \cap (-\infty ,-M)\times {\mathbb {R}}^+, \end{aligned}$$

which together with (95) implies (91), since

$$\begin{aligned} {\mathcal {H}}^1 \left( \partial \left( E_j \cap (-\infty , -M) \times {\mathbb {R}}^+\right) \cap (-\infty , -M)\times {\mathbb {R}}^+ \right) = |D \chi _{E_j}| ((-\infty , -M) \times {\mathbb {R}}^+), \end{aligned}$$

see (Ambrosio et al. (2000), Chapter 3.3). This concludes the proof of (vii).

**Step 2 (Approximation by polygons).** By Step 1, we can assume that $E \subset \subset B(R)$ is smooth and bounded. Furthermore, for such *E* we can construct a sequence of approximating polygons $P_{j}$ by choosing the vertices of each $P_{j}$ on the boundary of *E* in such a way that $|P_{j} \Delta E| \rightarrow 0$,

$$\begin{aligned} \int _{\partial P_{j} \cap (\mathbb {R}^2{\setminus }{\overline{S}}) } \Gamma (\nu _{P_{j}} ) d {\mathcal {H}}^1 \rightarrow \int _{\partial E \cap (\mathbb {R}^2{\setminus }{\overline{S}})} \Gamma (\nu _{E} ) d {\mathcal {H}}^1, \end{aligned}$$

and

$$\begin{aligned} {\mathcal {H}}^1 (\partial P_{j} \cap \partial S) \rightarrow {\mathcal {H}}^1 (\partial E \cap \partial S), \end{aligned}$$

so that

97 $$\begin{aligned} {\mathcal {E}} (P_{j}) \rightarrow {\mathcal {E}} (E). \end{aligned}$$

**Step 3 (Approximation by polygons with vertices on the lattice).** In view of previous steps and the metrizability of the unit ball of measures (where the norm is given by total variation) induced by the weak* convergence, by employing a standard diagonal argument and (97), we can assume, without loss of generality, that *E* has polygonal boundary. We now approximate such polygonal set *E*, whose number of vertices we denote by $m\in {\mathbb {N}}$ with a sequence of polygons $E_n$ characterized by *m* vertices belonging to

$$\begin{aligned} {\mathcal {L}}_F^n:=\frac{1}{\sqrt{n}} {\mathcal {L}}_F. \end{aligned}$$

More precisely, let $E_n$ be the polygon with vertices the set of *m* points in $\frac{1}{\sqrt{n}} {\mathcal {L}}_F$ closest in the Euclidean norm to the *m* vertices of *E*. Notice that the angles at the vertices of $E_n$ approximate the angles at the vertices of *E*, $|E_n \Delta E| \rightarrow 0$,

$$\begin{aligned} \int _{\partial E_{n} \cap (\mathbb {R}^2{\setminus }\overline{S_n}) } \Gamma (\nu _{E_{n}} ) d {\mathcal {H}}^1 \rightarrow \int _{\partial E \cap (\mathbb {R}^2{\setminus }{\overline{S}}) } \Gamma (\nu _{E} ) d {\mathcal {H}}^1 \end{aligned}$$

and

$$\begin{aligned}{\mathcal {H}}^1 (\partial E_{n} \cap \partial S_n ) \rightarrow {\mathcal {H}}^1 (\partial E \cap \partial S ). \end{aligned}$$

where $S_n$ is defined in (29). Therefore,

98 $$\begin{aligned} {\mathcal {E}}_{n} (E_n) \rightarrow {\mathcal {E}} (E), \end{aligned}$$

where ${\mathcal {E}}_{n}$ is defined in (28). Furthermore, there exist $\alpha _n\searrow 0$ and $\beta _n \searrow 0$ such that

99 $$\begin{aligned} \left| |E_n|-\frac{\sqrt{3}}{2}\right| =\alpha _n\qquad \text {and}\qquad |{\mathcal {H}}^1(\partial E_n)-{\mathcal {H}}^1(\partial E)|=\beta _n. \end{aligned}$$

We can assume that $\partial E_n \cap \partial S_n$ is a union of segments of length strictly greater than zero. Obviously, their number is bounded, independently on *n*.

**Step 4 (Discrete recovery sequence).** Let us now consider the sequence of crystalline configurations ${{\widetilde{D}}}_n :=\sqrt{n}({\mathcal {L}}_F^n \cap {\overline{E}}_n)$, and notice that $\mu _{{{\widetilde{D}}}_n}$ weakly* converges to $\rho \chi _E$. Furthermore, from the definition of scaled Voronoi cells *v*(*x*) of *x* (see (53)) it follows that

$$\begin{aligned} \#{{\widetilde{D}}}_n-n=\frac{2n}{\sqrt{3}}\sum _{x\in {{\widetilde{D}}}_n} |v(x)|\,-\,n=\frac{2n}{\sqrt{3}}\sum _{x\in \widetilde{D}_n{\setminus }\partial {{\widetilde{D}}}_n} |v(x)|\,-\,n\,+\,\frac{2n}{\sqrt{3}}\sum _{x\in \partial {{\widetilde{D}}}_n} |v(x)|, \end{aligned}$$

and hence, since for every $x\in {{\widetilde{D}}}_n$ we have $|v(x)| = \sqrt{3}/(2n)$,

100 $$\begin{aligned} |\#{{\widetilde{D}}}_n-n|&\le \frac{2}{\sqrt{3}}\alpha _nn + \#\partial {{\widetilde{D}}}_n \nonumber \\&\le \frac{2}{\sqrt{3}}\alpha _nn + C({\mathcal {H}}^1(\partial E) +\beta _n)\sqrt{n} \end{aligned}$$

for some constant $C>0$, where in the last inequality we used (99).

We now claim that

101 $$\begin{aligned} \frac{{{\widetilde{V}}}_n ({{\widetilde{D}}}_n)+6c_F \#\widetilde{D}_n}{\sqrt{n}}={\mathcal {E}}_n (E_n)+o(1), \end{aligned}$$

where ${{\widetilde{V}}}_n$ is the generalization of $V_n$ (see 10) to configurations with a number of atoms different than *n*, i.e.,

$$\begin{aligned} {{\widetilde{V}}}_n (D_k) := \sum _{i\ne j} v_{FF}(|d_i-d_j|)\,+\, \sum _{i=1}^k v^1(d_i), \end{aligned}$$

for every configuration $D_k:=\{d_1,\dots ,d_k\} \in {\mathcal {C}}_k$. The claim easily follows from the observation that each side $S_{k,n}$ of $E_n$, $k=1,\dots ,m$, for *n* large enough, intersects $\sqrt{n} \,(\Gamma (\nu _{S_{k,n}})/c_F) {\mathcal {H}}^1(S_{k,n})+O(1)$ segments such that $|z_1-z_2|=1/\sqrt{n}$ and $(z_1,z_2) \in {{\widetilde{D}}}_n/\sqrt{n} \times ({\mathcal {L}}_F^n \backslash ({{\widetilde{D}}}_n/\sqrt{n}))$ and from the observation that

102 $$\begin{aligned} \sum _{x \in \sqrt{n}\#{\widetilde{D}}_n} v^1(x)=-\frac{c_S}{q}{\mathcal {H}}^1(\partial E_n\cap \partial S_n)+o(1) \end{aligned}$$

To see the first observation, we begin by considering a segment $L=(x,y)$ with endpoints $x,y \in {\mathcal {L}}_F$. We denote the unit tangential and normal vector to *L* by ${{\varvec{t}}_L}$ and $\nu _{L}$, respectively. Obviously $y=x+{{\varvec{t}}}$ for the vector ${{\varvec{t}}}:={\mathcal {H}}^1(L) {{\varvec{t}}_L}=k_1 {\varvec{t}}_1+ k_2 {\varvec{t}}_2$ defined for some $k_1, k_2 \in {\mathbb {Z}}$. We restrict to the case in which $k_1,k_2 \in {\mathbb {N}}_0$ since the remaining case can be treated analogously. Let $\overline{\Gamma }$ be the function such that $\overline{\Gamma }({\varvec{t}_L})=\Gamma (\nu _L)/c_F$, i.e.,

$$\begin{aligned} \overline{\Gamma }({{\varvec{t}}_L}):= 2\left( {{\varvec{t}}_L^1} +\frac{{{\varvec{t}}_L^2}}{\sqrt{3}}\right) \end{aligned}$$

for

$$\begin{aligned} {{\varvec{t}}_L}:={{{\varvec{t}}_L^1} \atopwithdelims (){{\varvec{t}}_L^2}}, \end{aligned}$$

and extend $\overline{\Gamma }$ by homogeneity. Notice that

103 $$\begin{aligned} \overline{\Gamma }(k_1{\varvec{t}}_1)=2k_1 \qquad \text {and}\qquad \overline{\Gamma }(k_2{\varvec{t}}_2)=2k_2 . \end{aligned}$$

Let $P_L$ be the parallelogram with sides the vectors $x+k_1 {\varvec{t}}_1$ and $x+k_2{\varvec{t}}_2$. Furthermore, let $P_L^+$ and $P_L^-$ the open triangles in which *L* divides $P_L$. Notice that inside $P_L$ we have $k_1$ lines parallel to $k_2{\varvec{t}}_2$, $k_2$ lines parallel to $k_1{\varvec{t}}_1$, and $k_1+k_2-1$ lines with varying length that are parallel to the vector ${\varvec{t}}_2-{\varvec{t}}_1$. Since $x+{{\varvec{t}}}$ intersects each of these last lines (and each line intersects *L* one time), we have that *L* exactly intersects

$$\begin{aligned} 2(k_1+k_2)-1&= \overline{\Gamma }(k_1{\varvec{t}}_1)+\overline{\Gamma }(k_2{\varvec{t}}_2)-1=\overline{\Gamma }({\varvec{t}})-1\\&={\mathcal {H}}^1(L)\overline{\Gamma }({\varvec{t}_L})-1=\frac{{\mathcal {H}}^1(L)}{c_F}\Gamma (\nu _L) -1 \end{aligned}$$

lines and hence, *L* intersects ${\mathcal {H}}^1(L)\Gamma (\nu _L)/c_F$ segments $[z_1,z_2]$ such that $|z_1-z_2|=1$, $z_1\in {\mathcal {L}}_F\cap (P_L^+\cup L)$, and $z_2\in {\mathcal {L}}_F\cap P_L^-$.

Therefore, if we denote the *m* vertices of $$E_n$$ by $$v_{k,n}$$ for $$k=1,\dots ,m$$ and let $$v_{m+1,n}=v_{1,n}$$, then, for *n* large enough, each side $$S_{k,n}=[v_{k,n},v_{k+1,n}]$$ of $$E_n$$ intersects $$\sqrt{n} (\Gamma (\nu _{S_{k,n}})/c_F) {\mathcal {H}}^1(S_{k,n} )+O(1)$$ segments such that $$|z_1-z_2|=\frac{1}{\sqrt{n}}$$ and $$(z_1,z_2) \in {{\widetilde{D}}}_n \times ( \frac{1}{\sqrt{n}}{\mathcal {L}}_F \backslash \widetilde{D}_n)$$, where the contribution *O*(1) takes into account that the endpoints of $$S_{k,n} $$ might have a different numbers of neighbors in $${{\widetilde{D}}}_n$$. However, such disturbance is of the order *O*(1) since the angles of $$E_n$$ at the segment are approximately the same for all *n*. In the end, (102) easily follows from the fact that $$\partial E_n \cap \partial S_n$$ is a union of segments of length strictly greater than zero whose number is bounded, independently on *n*.

**Step 5 (Final recovery sequence).** Finally, we variate the configuration ${{\widetilde{D}}}_n$ to obtain configurations $D_n$ such that $\# D_n=n$. It can be easily seen that for every $m \in {\mathbb {N}}$ there exists a configuration *F*(*m*) that satisfies $\#F(m)=m$, $E_m (\mu _{F(m)})=O(\sqrt{m})$ and *F*(*m*) is a subset of a rhomb with side lengths $\lceil \sqrt{m}\, \rceil$.

If $\#{{\widetilde{D}}}_n<n$, we let $m:=n-\#{\widetilde{D}}_n$, properly translate *F*(*m*) so that it does not intersect ${\widetilde{D}}_n$, and we define

$$\begin{aligned}D_n:={{\widetilde{D}}}_n\cup F(m). \end{aligned}$$

By (100), there exists a constant $C>0$ such that

104 $$\begin{aligned} \left| \frac{{{\widetilde{V}}}_n ({{\widetilde{D}}}_n)+6c_F \#\widetilde{D}_n}{\sqrt{n}}-\frac{{{\widetilde{V}}}_n (D_n)+6c_F n}{\sqrt{n}} \right| \le C\frac{\sqrt{|\#{{\widetilde{D}}}_n-n|}}{\sqrt{n}} \rightarrow 0. \end{aligned}$$

Similarly, if $\#{{\widetilde{D}}}_n>n$, by (100) for *n* large enough we can define a configuration $D_n$ satisfying (104) by taking away $m= \#{{\widetilde{D}}}_n-n$ atoms from ${{\widetilde{D}}}_n$, for example, we can define $D_n:=\widetilde{D}_n{\setminus }F(m)$, for *F*(*m*) translated in a way that it and all its neighbors belong to the interior of $E_n$. Since $\mu _{D_n}$ weakly* converges to $\rho \chi _E$, the assertion follows from (101) and (104). $\square$

## Proof of the Main Theorems in the Dewetting Regime

We begin the section by stating a $\Gamma$-convergence results that is a direct consequence of Sects. 5 and 6. Recall that $\rho :=2/\sqrt{3}$.

### Theorem 7.1

($\Gamma$-convergence) Assume (32). The functional

105 $$\begin{aligned} E_n:= n^{-1/2}(I_n+6c_F n), \end{aligned}$$

where $I_n$ is defined by (16), $\Gamma$-converges with respect to the weak* convergence of measures to the functional $I_\infty$ defined by

106 $$\begin{aligned} I_\infty (\mu ):={\left\{ \begin{array}{ll} {\mathcal {E}}(D_\mu ), &{} \text {if } \exists D_\mu \subset \mathbb {R}^2{\setminus }S\text { set of finite perimeter}\\ &{} \quad \text {with }|D_\mu |=\frac{\sqrt{3}}{2}\text { such that } \mu =\frac{2}{\sqrt{3}}\chi _{D_\mu } \\ +\infty , &{}\text {otherwise,}\\ \end{array}\right. } \end{aligned}$$

for every $$\mu \in {\mathcal {M}}(\mathbb {R}^2)$$.

### Proof

In view of the definition of $\Gamma$-convergence the assertion directly follows from the lower and upper bound provided by Theorems 5.3 and  6.1, respectively. $\square$

We notice that Theorem 7.1 is not enough to conclude Assertion 3. of Theorem 2.4. In fact, the compactness provided for energy equi-bounded sequences $D_n\in {\mathcal {C}}_n$ by Theorem 4.4 of Sect. 4 holds only for almost-connected configurations $D_n$. Therefore, as detailed in the following result, we can deduce the convergence of a subsequence of minimizers only after performing (for example) the transformation ${\mathcal {T}}$ given by Definition 2.1, which does not change the property of being a minimizer.

### Corollary 7.2

Assume (32). For every sequence of minimizers $\mu _n\in {\mathcal {M}}_n$ of $E_n$, there exists a *(* possibly different*)* sequence of minimizers $\widetilde{\mu }_n\in {\mathcal {M}}_n$ of $E_n$ that admits a subsequence converging with respect to the weakly *convergence of measures to a minimizer of $I_\infty$ in

$$\begin{aligned} {\mathcal {M}}_W \,:=&\,\bigg \{\mu \in {\mathcal {M}}(\mathbb {R}^2)\ :\ \exists D\subset \mathbb {R}^2{\setminus }S\text { set of finite perimeter,} \text { bounded, } \\&\text { with }|D|=\frac{1}{\rho },\text { and such that } \mu =\rho \chi _D\bigg \}. \end{aligned}$$

### Proof

Let $\mu _n\in {\mathcal {M}}_n$ be minimizers of $E_n$. By (15), (16), and (105) there exist configurations $D_n\in {\mathcal {C}}_n$ such that $\mu _n:=\mu _{D_n}$. Let ${\mathcal {T}}(D_n)\in {\mathcal {C}}_n$ be the transformed configurations associated with $D_n$ by Definition 2.1. We notice that the sequence of measures

$$\begin{aligned} {\widehat{\mu }}_n:=\mu _{{\mathcal {T}}(D_n)} \end{aligned}$$

is also a sequence of minimizers of $E_n$, since by Definition 2.1 and (17) we have that

$$\begin{aligned} E_n({\widehat{\mu }}_n)\le E_n(\mu _n). \end{aligned}$$

Therefore, by Theorems 7.1 and 4.4 we obtain that there exist a sequence of vectors $a_n:=t_n{\varvec{t}}_1$ for $t_n\in \mathbb {Z}$, an increasing sequence $n_k$, $k\in \mathbb {N}$, and a measure $\mu \in {\mathcal {M}}_W$ (being a minimizer of $I_{\infty }$) such that $\widetilde{\mu }_{n_k}\rightharpoonup ^*\mu$ in ${\mathcal {M}}(\mathbb {R}^2)$, where

$$\begin{aligned} {\widetilde{\mu }}_n:={\widehat{\mu }}_n(\cdot +a_{n}). \end{aligned}$$

This concludes the proof. $\square$

In view of Theorem 2.3, we can improve the previous result and in turns, prove the convergence of minimizers (up to a subsequence) directly without passing to an auxiliary sequence of minimizers obtained by performing the transformation ${\mathcal {T}}$ given by Definition 2.1. In fact, Theorem 2.3 allows to exclude the possibility that a sequence of (not almost-connected) minimizers $\mu _n\in {\mathcal {M}}_n$ loses mass in the limit.

### Proof of Theorem 2.3

Let ${\widehat{D}}_n$ be such that

$$\begin{aligned} V_n({\widehat{D}}_n)=\min _{D_n\in {\mathcal {C}}_n}{V_n(D_n)}, \end{aligned}$$

and select for every ${\widehat{D}}_n$ a connected component ${\widehat{D}}_{n,1}\subset {\widehat{D}}_n$ with largest cardinality.

We assume by contradiction that

$$\begin{aligned} \liminf _{n \rightarrow \infty } \mu _{{{\widehat{D}}}_{n}} ({{\widehat{D}}}_{n,1})<1, \end{aligned}$$

and we select a subsequence $n_k$ such that

107 $$\begin{aligned} \lim _{k \rightarrow \infty } \mu _{{{\widehat{D}}}_{n_k}} (\widehat{D}_{n_k,1})=\liminf _{n \rightarrow \infty } \mu _{{{\widehat{D}}}_{n}} (\widehat{D}_{n,1})<1. \end{aligned}$$

By Corollary 7.2, there exists a (possibly different) sequence of minimizers $\widetilde{\mu }_{n_k}\in {\mathcal {M}}_{n_k}$ of $E_{n_k}$ that (up to passing to a non-relabeled subsequence) converge with respect to the weak* convergence of measures to a minimizer $\mu \in {\mathcal {M}}_W$ of $I_\infty$. Therefore, there exists a bounded set $D\subset \mathbb {R}^2{\setminus }S$ of finite perimeter with $|D|=1/\rho$ such that $\mu = \rho \chi _D$ and ${\widetilde{\mu }}_{n_k}$ converge with respect to the weak* convergence to $\rho \chi _D$.

We claim that

108 $$\begin{aligned} m_0:={\mathcal {E}}(D)>0, \end{aligned}$$

and observe that (108) follows from

109 $$\begin{aligned} \int _{\partial ^* D \cap \{x_2>0\}} \Gamma (\nu _{D} ) d {\mathcal {H}}^1 \ge 2c_F{\mathcal {H}}^1(\partial ^*D \cap \{x_2=0\}). \end{aligned}$$

In order to prove (109), we first show that

110 $$\begin{aligned} \int _{\partial ^* D} \nu _D d {\mathcal {H}}^1=0, \end{aligned}$$

by taking $\varphi _i \in C_c^{1}({\mathbf {R}}^2;{\mathbf {R}}^2)$, $\varphi _i={\varvec{t}}_i$ on some open set *F* such that $D\subset \subset F$, for $i=1,3$, in the identity

$$\begin{aligned} \int _{\partial ^* D} \langle \nu _D,\varphi _i \rangle d {\mathcal {H}}^1= - \int _{D}\text {div} \varphi _i dx =-\int _{D}\text {div} {\varvec{t}}_idx =0, \end{aligned}$$

where we used the definition of reduced boundary and the *generalized Gauss–Green formula* (Ambrosio et al. 2000, Theorem 3.36) for sets of finite perimeter. Then, from (110) it follows that

$$\begin{aligned} \int _{\partial ^* D \cap \{x_2>0\}} \nu _D d{\mathcal {H}}^1 ={\mathcal {H}}^1 (\partial ^* D \cap \{x_2=0\}){\varvec{t}}_3\end{aligned}$$

and hence, since $\Gamma$ is convex and homogeneous, by Jensen’s inequality and the fact that $\Gamma ({\varvec{t}}_3)=2c_F$ we conclude that

$$\begin{aligned} \int _{\partial ^* D \cap \{x_2>0\}} \Gamma (\nu _{D} ) d {\mathcal {H}}^1 \ge L \Gamma \left( \frac{1}{L}\int _{\partial ^*`D \cap \{x_2>0\}}\nu _{D} d{\mathcal {H}}^1 \right) = 2c_F{\mathcal {H}}^1 (\partial ^* D \cap \{x_2=0\}), \end{aligned}$$

where $L:={\mathcal {H}}^1(\partial ^*D \cap \{x_2>0\})$, which is (109).

We claim that there exist configurations $\widetilde{D}_{n_k}\in {\mathcal {C}}_{n_k}$ defined by

$$\begin{aligned} {{\widetilde{D}}}_{n_k}:={{\widetilde{D}}}_{n_k}^1\cup {{\widetilde{D}}}_{n_k}^2, \end{aligned}$$

where ${{\widetilde{D}}}_{n_k}^1$ and ${{\widetilde{D}}}_{n_k}^2$ are configurations such that:

(i) $\text {supp } \mu _{{{\widetilde{D}}}_{n_k}^1}\subset B(x_1,R_1)$ and $\text {supp } \mu _{{{\widetilde{D}}}_{n_k}^1}\subset B(x_2,R_2)$ for some $x_1,x_2\in \mathbb {R}^2$ and $R_1,R_2>0$ with $B(x_1,R_1) \cap B(x_2,R_2)=\emptyset$,
(ii) the energy is preserved, i.e.,
  $$\begin{aligned} V_{n_k}({{\widetilde{D}}}_{n_k})=V_{n_k}({{\widehat{D}}}_{n_k}), \end{aligned}$$
(iii) the following inequalities hold:
  $$\begin{aligned} \liminf _{k \rightarrow \infty } \mu _{{{\widetilde{D}}}_{n_k}}(\widetilde{D}_{n_k}^1)>0 \quad \text {and}\quad \liminf _{k \rightarrow \infty } \mu _{{{\widetilde{D}}}_{n_k}}({{\widetilde{D}}}_{n_k}^2)>0. \end{aligned}$$

Under the further assumption that

111 $$\begin{aligned} \lim _{k \rightarrow \infty } \mu _{{{\widehat{D}}}_{n_k}} ({{\widehat{D}}}_{n_k,1})>0, \end{aligned}$$

we can explicitly define $\widetilde{D}_{n_k}^1:={\mathcal {T}}({\widehat{D}}_{n_k} \backslash {\widehat{D}}_{n_k,1})$ and ${{\widetilde{D}}}_{n_k}^2:=\widehat{D}_{n_k,1}+tq{\varvec{t}}_1$ for some large $t\in \mathbb {Z}$ (see (13) for the definition of *q*). In fact, the configurations $\widetilde{D}_{n_k}^1$ and ${{\widetilde{D}}}_{n_k}^2$ are bounded because by Definition 2.1 they are almost connected and hence, property (i) is satisfied provided that $t\in \mathbb {Z}$ is chosen large enough. Furthermore, again by Definition 2.1 (and the translation of ${{\widehat{D}}}_{n_k,1}$ of *q*-multiples) property (ii) is verified. Finally, property (iii) directly follows from (107) and (111).

If condition (111) is not satisfied, the definition of ${{\widetilde{D}}}_{n_k}^1$ and ${{\widetilde{D}}}_{n_k}^2$ is more involved. We choose an order among the connected components of $\widehat{D}_{n_k}$ other than ${{\widehat{D}}}_{n_k,1}$, say $\widehat{D}_{n_k,\ell }$ for $\ell \ge 2$ with the convention that $\widehat{D}_{n_k,\ell }:=\emptyset$ for $\ell$ larger than the number of connected components of ${{\widehat{D}}}_{n_k}$, and we observe that

$$\begin{aligned} 0\le \lim _{k \rightarrow \infty } \max _{l \in {\mathbb {N}}} \mu _{\widehat{D}_{n_k}} ({{\widehat{D}}}_{n_k,\ell })\le \lim _{k \rightarrow \infty } \mu _{{{\widehat{D}}}_{n_k}} ({{\widehat{D}}}_{n_k,1})=0, \end{aligned}$$

so that

112 $$\begin{aligned} \lim _{k \rightarrow \infty } \max _{l \in {\mathbb {N}}} \mu _{{{\widehat{D}}}_{n_k}} ({{\widehat{D}}}_{n_k,\ell })=0. \end{aligned}$$

Furthermore, from (112) and the fact that

113 $$\begin{aligned} \mu _{{{\widehat{D}}}_{n_k}}\left( \bigcup _\ell \widehat{D}_{n_k,\ell }\right) =1, \end{aligned}$$

it follows that there exist $J_k\ge 2$ such that

114 $$\begin{aligned} \sum _{j=1}^{J_k-1}\mu _{{{\widehat{D}}}_{n_k}} (\widehat{D}_{n_k,\ell _j})\le \frac{1}{3} \quad \text {and}\quad \sum _{j=1}^{J_k}\mu _{{{\widehat{D}}}_{n_k}} (\widehat{D}_{n_k,\ell _j})>\frac{1}{3}. \end{aligned}$$

We define

$$\begin{aligned} {\widetilde{D}}_{n_k}^1:={\mathcal {T}}\left( \bigcup _{j=1}^{J_k}\widehat{D}_{n_k,\ell _j}\right) \quad \text {and}\quad \widetilde{D}_{n_k}^2:={\mathcal {T}}\left( {\widehat{D}}_{n_k} \backslash \left( \bigcup _{j=1}^{J_k}\widehat{D}_{n_k,\ell _j}\right) \right) +t'q{\varvec{t}}_1\end{aligned}$$

for a large $t'\in \mathbb {N}$. As in the previous case properties (i) and (ii) directly follow from Definition 2.1 and the choice of the ${\varvec{t}}_1$-translation by a *q*-multiple $t'\in \mathbb {N}$ large enough, where *q* is defined in (13). Finally, property (iii) is also satisfied since

$$\begin{aligned}&\liminf _{k \rightarrow \infty } \mu _{{{\widetilde{D}}}_{n_k}}({{\widetilde{D}}}_{n_k}^2)\\&\quad \ge \lim _{k \rightarrow \infty } \mu _{\widehat{D}_{n_k}}\left( \bigcup _{\ell \in \mathbb {N}} {{\widehat{D}}}_{n_k,\ell }\right) - \limsup _{k \rightarrow \infty } \mu _{\widetilde{D}_{n_k}}\left( \bigcup _{j=1}^{J_k-1}{{\widehat{D}}}_{n_k,\ell _j}\right) - \lim _{k \rightarrow \infty } \mu _{{{\widehat{D}}}_{n_k}} ({{\widehat{D}}}_{n_k,J_k})\\&\quad =1- \limsup _{k \rightarrow \infty } \mu _{\widetilde{D}_{n_k}}\left( \bigcup _{j=1}^{J_k-1}{{\widehat{D}}}_{n_k,\ell _j}\right) -0\ge \frac{2}{3}>0, \end{aligned}$$

where we used (113) and (112). Therefore, the claim is verified.

By such claim and the same arguments used in Theorem 4.4, we deduce that (up to a non-relabeled subsequence)

$$\begin{aligned} \mu _{{{\widetilde{D}}}_{n_k}^j}\rightharpoonup ^*\frac{1}{|D^j|}\chi _{D^j} \end{aligned}$$

in ${\mathcal {M}}(\mathbb {R}^2)$ for $j=1,2$, with $D^j$ disjoint bounded sets of finite perimeter such that

$$\begin{aligned} D'=D^1\cup D^2, \end{aligned}$$

where $D'$ is a minimizer of ${\mathcal {E}}$. Therefore, if with $\lambda _j:=|D^j|$, then

115 $$\begin{aligned} \lambda _1+\lambda _2=|D'| =\frac{\sqrt{3}}{2} \end{aligned}$$

with both $\lambda _1>0$ and $\lambda _2>0$, respectively, because of (i) and (iii) above. Finally, by scaling arguments we conclude

$$\begin{aligned} m_0={\mathcal {E}}(D)={\mathcal {E}}(D^1)+{\mathcal {E}}(D^2)= \sqrt{\frac{2}{\sqrt{3}}} \left( \sqrt{\lambda _1} m_0 +\sqrt{\lambda _2} m_0\right) \end{aligned}$$

and hence, by (108) and (115) we obtain

$$\begin{aligned} \sqrt{\lambda _1} +\sqrt{\lambda _2}=\sqrt{\sqrt{3}/2}=\sqrt{\lambda _1+\lambda _2}, \end{aligned}$$

which implies $\lambda _1=0$ or $\lambda _2=0$ that is a contradiction. $\square$

We are now ready to prove Theorem 2.4.

### Proof of Theorem 2.4

Assertions 1. and 2. directly follow from Theorem 7.1 and Corollary 7.2, respectively. It remains to show Assertion 3. to which the rest of the proof is devoted.

Let $\mu _n\in {\mathcal {M}}_n$ be minimizers of $E_n$. By Corollary 7.2, there exist another sequence of minimizers ${\widetilde{\mu }}_n\in {\mathcal {M}}_n$ of $E_n$, an increasing sequence $n_k$ for $k\in \mathbb {N}$, and a measure $\mu \in {\mathcal {M}}_W$ minimizing $I_\infty$ such that

116 $$\begin{aligned} {\widetilde{\mu }}_{n_k}\rightharpoonup ^*\mu \end{aligned}$$

in ${\mathcal {M}}(\mathbb {R}^2)$. In particular, from the proof of Corollary 7.2 we observe that

$$\begin{aligned} {\widetilde{\mu }}_n:=\mu _{{\mathcal {T}}(D_n)}(\cdot +t_{n}{\varvec{t}}_1) \end{aligned}$$

for some integers $t_n\in \mathbb {Z}$, and for configurations $D_n\in {\mathcal {C}}_n$ such that $\mu _n:=\mu _{D_n}$, where ${\mathcal {T}}(D_n):={\mathcal {T}}_2({\mathcal {T}}_1(D_n))$ (see Definition 2.1). Furthermore, by (16) and (105) the configurations $D_n$ are minimizers of $V_n$ in ${\mathcal {C}}_n$ and hence, ${\mathcal {T}}_1(D_n)=D_n$ and by Theorem 2.3 we have that, up to a non-relabeled subsequence,

117 $$\begin{aligned} \lim _{k \rightarrow \infty } \mu _{D_{n_k}} (D_{{n_k},1})=1, \end{aligned}$$

where $D_{{n_k},1}$ is a connected component of $D_{n_k}$ (with the largest cardinality). We also observe that the transformation ${\mathcal {T}}_2$ consists in translations of the connected components of $D_{n_k}$ with respect to a vector in the direction $-{\varvec{t}}_1$ with norm (depending on the component) in $\mathbb {N}\cup \{0\}$. Let $t'_{n_k}\in \mathbb {N}\cup \{0\}$ be the norm of the vector for the connected component $D_{{n_k},1}$. From (116) and (117), it follows that

$$\begin{aligned} \mu _{D_{n_k}}(\cdot +(t_{n_k}-t'_{n_k}){\varvec{t}}_1)\rightharpoonup ^*\mu \end{aligned}$$

and hence, we can choose $c_{n_k}:=t_{n_k}-t'_{n_k}\in \mathbb {Z}$. $\square$

## Examples of Other Positioning of Reference Lattices

The analysis presented in this manuscript is to be intended as a first attempt to model crystalline drops on rigid substrates without the ambition of directly provide a comprehensive treatment. We aim at introducing a specific mathematical setting, which could be a reference for further developments and already incorporates techniques useful for more general cases. In particular, the results presented in the previous sections relate to a particular positioning of the film and the substrate reference lattice that depends on the definition of $x_F$ in (7) chosen to be equal to

$$\begin{aligned} x_F^0:=(0,e_{FS}) \end{aligned}$$

by (8). Such positioning might not be energetically optimal in certain situations, meaning for example that at the discrete level for specific choices of the *vector of parameters* used in the mathematical setting introduced in Sect. 2, which we denote in the following as

$$\begin{aligned} \Lambda ^0:=(e_F,e_{FS},e_{S},c_F,c_S), \end{aligned}$$

the same drop configurations could have a lower energy for some other choices of $x_F\in \mathbb {R}^2{\setminus }{\overline{S}}$.

We do not intend to address here the general case of all possible positionings for a fixed reference film lattice, as this requires a too lengthy and involved treatment, which is the subject of Piovano and Velčić (2021) and of the forthcoming paper (Piovano and Velčić in preparation). However, we would like to mention that the model considered in this current paper will be one of the very few necessary settings to which the greater generality of positionings of film and substrate reference lattices considered in Piovano and Velčić (2021) and Piovano and Velčić (in preparation) is reduced, and we conclude the paper by presenting in this section some relevant settings where the optimal positioning is different, but can be easily reduced to the model of Sect. 2.

To this end, we denote in the following by ${\mathcal {M}}_{\Lambda }(x_F)$ the model analogous to the model introduced in Sect. 2 where $x_F:=(x_{F,1},x_{F,2})$ referred to as the *center of the film lattice* is free to be fixed in any point in $\mathbb {R}^2{\setminus }{\overline{S}}$ and $\Lambda$ is any admissible *vector of parameters*, i.e.,

$$\begin{aligned} \Lambda :=(e_F',e_{FS}',e_{S}',c_F',c_S'). \end{aligned}$$

More precisely, we make explicit the dependence on $x_F:=(x_{F,1},x_{F,2})$ and $\Lambda$ in the choice of

$$\begin{aligned} {\mathcal {L}}_{F,\Lambda }(x_F):= & {} \{x_F+k_1 {\varvec{t}}_1+k_2 {\varvec{t}}_2\,:\, k_1\in \mathbb {Z}\text { and }k_2\in \mathbb {N}\cup \{0\}\}, \\ \partial {\mathcal {L}}_{S,\Lambda }:= & {} \{s_k:=(k e_S,0) \,:\,k\in \mathbb {Z}\}, \end{aligned}$$

and the related family of configurations

$$\begin{aligned} {\mathcal {C}}_{n,\Lambda }(x_F):= \{A\subset {\mathcal {L}}_{F,\Lambda }(x_F)\,:\, \#A=n\} \end{aligned}$$

and denote the discrete energy of any configuration $D_n\in {\mathcal {C}}_{n,\Lambda }(x_F)$ by $V_{n,\Lambda ,x_F}(D_n)$ (for $v_{F\alpha }$ defined as in (9) with respect to the parameters in $\Lambda$).

We observe that the specific model defined in Sect. 2 corresponds to the model ${\mathcal {M}}_{\Lambda ^0}(x_F^0)$ (with $\Lambda ^0:=(e_F,e_{FS},e_S,c_F,c_S)$ for which we recall that $e_F$ was normalized to 1) with family of configurations ${\mathcal {C}}_{n,\Lambda ^0}(x_F^0)$, which we indicate for simplicity in the following with

$$\begin{aligned} {\mathcal {M}}^0:={\mathcal {M}}_{\Lambda ^0}(x_F^0). \end{aligned}$$

For ${\mathcal {M}}^0$ we keep on using the same notation of previous sections for the family of configurations and the discrete energy related to ${\mathcal {M}}^0$, namely

$$\begin{aligned} {\mathcal {C}}_n:={\mathcal {C}}_{n,\Lambda ^0}(x_F^0) \end{aligned}$$

and

$$\begin{aligned} V_{n}:= V_{n,\Lambda ^0,x_F^0} \end{aligned}$$

in ${\mathcal {C}}_n$, respectively. We also recall that *q* and *p* are the natural numbers as defined in (13) such that

$$\begin{aligned} e_{S}=\frac{q}{p}. \end{aligned}$$

We now introduce the notion of *equivalent configurations* to ${\mathcal {M}}^0$ among the configurations in the various families ${\mathcal {C}}_{n,\Lambda }(x_F)$ defined for different $x_F\in \mathbb {R}^2{\setminus }{\overline{S}}$.

### Definition 8.1

Given ${\mathcal {M}}_{\Lambda }(x_F)$ and a vector of parameter $\Lambda :=(e_F',e_{FS}',e_S',c_F',c_S')$, we say that $D_n^0$ is the *associated configuration* in ${\mathcal {C}}_n^0$ of a configuration $D_n\in {\mathcal {C}}_{n,\Lambda }(x_F)$ if and only if

$$\begin{aligned} D_n^0:=D_n-x_F+x_F^0. \end{aligned}$$

Furthermore, we say that the model ${\mathcal {M}}_\Lambda (x_F)$ is *equivalent* to ${\mathcal {M}}^0$ if

$$\begin{aligned} V_{n,\Lambda ,x_F}(D_n)=V_{n}(D_n^0) \end{aligned}$$

for every $$D_n\in {\mathcal {C}}_{n,\Lambda }(x_F)$$.

The following definition allows to compare two models ${\mathcal {M}}_{\Lambda }(x_F)$ with different centers $x_F$ of the film lattice and vectors of parameters $\Lambda$.

### Definition 8.2

For $k=1,2$ let $x_F^k\in \mathbb {R}^2{\setminus }{\overline{S}}$ and let $\Lambda ^k$ be admissible vectors of parameters. We denote ${\mathcal {M}}^k:={\mathcal {M}}_{\Lambda ^k}(x_F^k)$, ${\mathcal {C}}_{n}^k:={\mathcal {C}}_{n,\Lambda ^k}(x_F^k)$, and $V_n^k:= V_{n,\Lambda ^k,x_F^k}$, and say that the model ${\mathcal {M}}^1$ has a (*energetically*) *better positioning of the reference lattices* than the model ${\mathcal {M}}^2$ if

$$\begin{aligned} V_n^1(D_n)\le V_n^2(D_n-x_F^1+x_F^2) \end{aligned}$$

for every $D_n\in {\mathcal {C}}_n^1$ and there exists $D_n'\in {\mathcal {C}}_n^1$ such that $V_n^1(D_n') < V_n^2(D_n'-x_F^1+x_F^2)$. If neither of ${\mathcal {M}}^1$ and ${\mathcal {M}}^2$ have a *better positioning*, then we say that ${\mathcal {M}}^1$ and ${\mathcal {M}}^2$ are *not comparable*.

With the following proposition, we recover the same results obtained for ${\mathcal {M}}^0$ for every model equivalent to ${\mathcal {M}}^0$.

### Proposition 8.3

For every model ${\mathcal {M}}_{\Lambda }(x_F)$ equivalent to ${\mathcal {M}}^0$ in the sense of Definition 8.1 for some $x_F\in \mathbb {R}^2{\setminus }{\overline{S}}$, and vector of parameters $\Lambda$ all the main results of Sect. 7, i.e., Theorems 2.2, 2.3, and 2.4, remain valid for ${\mathcal {M}}_{\Lambda }(x_F)$.

### Proof

The assertion follows by simply observing that all results of Sect. 7 are valid for the family of associated configurations $D_n^0$ of the configurations $D_n\in {\mathcal {C}}_{n,\Lambda }(x_F)$, and that $V_{n,\Lambda ,x_F}(D_n)=V_n(D_n^0)$ since ${\mathcal {M}}_{\Lambda }(x_F)$ is equivalent to ${\mathcal {M}}^0$. Therefore, the same wetting condition (31), the corresponding dewetting condition (32), and the same form (26) for the limiting energy ${\mathcal {E}}$ are obtained also for ${\mathcal {M}}_{\Lambda }(x_F)$. $\square$

We now list some relevant examples of models ${\mathcal {M}}_{\Lambda }(x_F)$ for $\Lambda :=(e_F',e_{FS}',e_S',c_F',c_S')$ and $x_F\in \mathbb {R}^2{\setminus }{\overline{S}}$ that are equivalent to ${\mathcal {M}}_0$ in the sense of Definition 8.1.

We begin with an example in which the positioning of $x_F$ allows each film atom in $\partial {\mathcal {L}}_{F,\Lambda }(x_F)$ to be connected with exactly two (neighboring) substrate atoms, providing an optimal positioning for film atoms, that is equivalent to ${\mathcal {M}}^0$ and hence, for which Proposition 8.3 holds.

### Example 8.4

If $q=1$ and $e_{S}=e_{FS}=1/p$, then the model ${\mathcal {M}}_{\Lambda }(x_F)$ defined for

$$\begin{aligned} x_F:=\frac{e_S}{2}\left( 1,\sqrt{3}\right) , \end{aligned}$$

with $e'_{FS}=e'_{S}:=e_S$, $c_F':=c_F$, and $c_S':=2c_S$, is equivalent to ${\mathcal {M}}^0$.

The following is an example of a model in which every substrate atom can be bonded to two film atoms that has a better positioning than the corresponding model with same vector of parameters and center in $x_F^0$, but that can also be reduced to ${\mathcal {M}}^0$.

### Example 8.5

If $q=p=1$ and $e_{S}=e_{FS}=1$, then the model ${\mathcal {M}}_{\Lambda }(x_F^1)$ defined with

$$\begin{aligned} x_F^1:=\frac{1}{2}\left( 1,\sqrt{15}\right) , \end{aligned}$$

$e'_S=e'_{FS}:=2$, $c'_{F}:=c_F$, and $c'_{S}:=c_S$ is equivalent to ${\mathcal {M}}^0$. We notice that every film atom in $\partial {\mathcal {L}}_{F,\Lambda }(x_F^1)$ is connected with exactly one substrate atom and that every substrate atom is bonded with exactly two film atoms in $\partial {\mathcal {L}}_{F,\Lambda }(x_F^1)$. Regarding the optimality among lattice positioning of ${\mathcal {M}}_{\Lambda }(x_F^1)$ we can observe that ${\mathcal {M}}_{\Lambda }(x_F^1)$ has, e.g., a better positioning than ${\mathcal {M}}_{\Lambda }(x_F^0)$, but that there is a model ${\mathcal {M}}_{\Lambda }(x_F^2)$ which is not comparable with ${\mathcal {M}}_{\Lambda }(x_F^1)$, e.g., choose

$$\begin{aligned} x_F^2:=\left( 1,\sqrt{3}\right) , \end{aligned}$$

despite the fact that in the model ${\mathcal {M}}_{\Lambda }(x_F^2)$ every second atom of $\partial {\mathcal {L}}_{F,\Lambda }(x_F^2)$ can be bonded with two substrate atoms. Notice also that the model ${\mathcal {M}}_{\Lambda }(x_F^2)$ defined in the previous example represents a case in which the configurations in ${\mathcal {C}}_{n,\Lambda }(x_F^2)$ with finite energy $V_{n,\Lambda ,x_F^2}$ need to have every second lattice site in $\partial {\mathcal {L}}_{F,\Lambda }(x_F^2)$ free of atoms (see the discussion after Example 8.6 for further aspects of ${\mathcal {M}}_{\Lambda }(x_F^2)$).

We discuss one more example that is equivalent to model ${\mathcal {M}}^0$ for which we have the particular situation in which every substrate atom can be bonded with exactly one film atom apart from periodically each third of them, which cannot be bonded with film atoms.

### Example 8.6

If $q=p=1$ and $e_{FS}=e_S=1$, then the model ${\mathcal {M}}_{\Lambda }(x_F^1)$ defined with

$$\begin{aligned} x_F^1:=\frac{1}{6}\left( -1,\sqrt{15}\right) , \end{aligned}$$

$e'_S=e'_{FS}:=2/3$, $c'_{F}:=c_F$, and $c'_{S}:=c_S$ is equivalent to ${\mathcal {M}}^0$. Every film atom in $\partial {\mathcal {L}}_{F,\Lambda }(x_F^1)$ is bonded with exactly one substrate atom, and every substrate atom can be bonded with exactly one film atom apart from periodically each third of them, which cannot be bonded with film atoms. We notice that this model has a better positioning than the model ${\mathcal {M}}_{\Lambda }(x_F^0)$ and is not comparable with the model ${\mathcal {M}}_{\Lambda }(x_F^2)$, where

$$\begin{aligned} x_F^2:=\frac{1}{3}\left( 1,\sqrt{3}\right) , \end{aligned}$$

despite the fact that in the model ${\mathcal {M}}_{\Lambda }(x_F^2)$ every second atom of $\partial {\mathcal {L}}_{F,\Lambda }(x_F^2)$ can be bonded with two substrate atoms, since it is also a case in which the configurations in ${\mathcal {C}}_{n,\Lambda }(x_F^2)$ with finite energy $V_{n,\Lambda ,x_F^2}$ need to have every second lattice site in $\partial {\mathcal {L}}_{F,\Lambda }(x_F^2)$ free of atoms.

We notice that Examples 8.5 and 8.6 also shows that models in which certain sites of the lower border of the film lattice are prevented for configurations with finite energy (i.e., the settings denoted by ${\mathcal {M}}_{\Lambda }(x_F^2)$ in both examples) do not present in principle a better positioning than models equivalent to ${\mathcal {M}}^0$, despite that the other (allowed) film atoms on the film-lattice border have two bonds with substrate atoms. For these settings which cannot be reduced to ${\mathcal {M}}^0$ we prove in the forthcoming paper (Piovano and Velčić in preparation) by considering a modification of the model ${\mathcal {M}}^0$ that analogous results to the ones contained in Sect. 7 hold true, but with different wetting condition and adhesivity parameter $\sigma$ for the limiting energy of the type (26).

Also the model ${\mathcal {M}}_{\Lambda } (x_F)$ defined by

$$\begin{aligned} x_F:=\frac{1}{8}\left( -1,\sqrt{35}\right) , \end{aligned}$$

$e'_S=e'_{FS}:=3/4$, $e_F'=1$, and by any admissible $c_F'$ and $c_S'$, will be reduced in Piovano and Velčić (in preparation) to a modification of ${\mathcal {M}}^0$ for which similar arguments allow us to prove analogous results to the ones contained in Sect. 7, but with wetting condition replaced by $c_S' \ge 5c_F'$ and adhesivity parameter in the limiting energy (26) given by

$$\begin{aligned} \sigma = 2c_F'-\frac{2}{3}c_S'. \end{aligned}$$

Such a model is interesting since every third film atom in $\partial {\mathcal {L}}_{F,\Lambda }(x_F)$ cannot be bonded with any substrate atom and every other film atom in $\partial {\mathcal {L}}_{F,\Lambda }(x_F)$ is connected with exactly one substrate atom. Furthermore, there exist substrate atoms in $\partial {\mathcal {L}}_{S,\Lambda }$ that are not connected with any film atoms. We also notice that this model has better positioning than the model ${\mathcal {M}}_{\Lambda }(x_F^0)$ since in the period of three film atoms in $\partial {\mathcal {L}}_{F,\Lambda }(x_F)$ two neighboring film atoms are connected with one substrate atom, while in $\partial {\mathcal {L}}_{F,\Lambda }(x_F^0)$ only one.
